# Pregnancy exacerbates neutrophil responses in murine lungs and alters gut microbiota composition after cigarette smoke exposure

**DOI:** 10.3389/fimmu.2025.1590290

**Published:** 2025-08-11

**Authors:** Ali Dehghani, Lei Wang, Johan Garssen, Eirini Styla, Thea Leusink-Muis, Ingrid van Ark, Gert Folkerts, Jeroen van Bergenhenegouwen, Saskia Braber

**Affiliations:** ^1^ Division of Pharmacology, Utrecht Institute for Pharmaceutical Sciences, Faculty of Science, Utrecht University, Utrecht, Netherlands; ^2^ Outpatient Clinic for Occupational Medicine, Department of Public and Occupational Health, Amsterdam UMC, Amsterdam, Netherlands; ^3^ Department of Pathology and Medical Biology, University Medical Center Groningen, University of Groningen, Groningen, Netherlands; ^4^ Danone Research and Innovation, Utrecht, Netherlands

**Keywords:** pregnancy, air pollution, environmental tobacco smoke, neutrophil chemotaxis, gut microbiota, lung transcriptomics

## Abstract

**Introduction:**

Air pollution, particularly environmental tobacco smoke, poses significant health risks, especially to pregnant women and their infants. This study explores the difference in response to cigarette smoke (CS) exposure between pregnant and non-pregnant mice by examining lung transcriptomic profiles, neutrophil numbers, key mediators of neutrophil chemotaxis, and gut microbiota composition.

**Methods:**

Pregnant and non-pregnant mice were exposed to either air or CS. Bronchoalveolar lavage fluid (BALF) was analyzed for inflammatory cells and mediators. RNA sequencing was conducted on lung tissue to identify transcriptomic alterations. Gut microbiota composition and short-chain fatty acid (SCFA) levels were assessed to explore the interactions within the gut-lung axis.

**Results:**

CS exposure resulted in a significant increase in inflammatory cells in the BALF, notably neutrophils, with pregnant dams showing a more substantial increase compared to non-pregnant mice. Transcriptomic analysis revealed neutrophil chemotaxis as the most enriched pathway in CS-exposed pregnant dams. Key genes associated with neutrophil-mediated inflammation, such as CXCL1, S100A8, and S100A9, were significantly upregulated. Gut microbiota analysis showed altered composition and reduced alpha and beta diversity in CS-exposed pregnant dams compared with air-exposed pregnant dams, along with compositional differences between CS-exposed pregnant and non-pregnant mice. CS exposure also resulted in a decrease in cecal SCFA levels in pregnant dams.

**Discussion:**

In conclusion, pregnancy as well as CS exposure induce differences in lung transcriptomic responses which might drive exacerbated lung inflammatory responses measured as neutrophil influx and activity. Microbiota functional and compositional states are also affected by both pregnancy and CS exposure, possibly indicating a gut-lung bidirectional effect.

## 1 Introduction

Air pollution, both indoor and outdoor, is increasingly recognized as a significant determinant of adverse health outcomes, particularly in vulnerable populations such as pregnant women and neonates (1–3). According to the World Health Organization (WHO), 99% of the world’s population breathe air that exceeds WHO air quality limits, containing high levels of pollutants (4).

Environmental tobacco smoke (ETS or secondhand smoking) is a major source of indoor air pollution and is globally recognized as a risk factor for both acute and chronic respiratory illnesses, contributing to over 800,000 premature deaths annually as part of the global tobacco epidemic (3, 5, 6). ETS, as well as cigarette smoke, is an extremely complex and dynamic mixture containing more than 7,000 chemicals and carcinogens (6, 7).

In addition to exposure to ETS (8), smoking during pregnancy is still prevalent in many parts of the world, with rates varying from 5.9% in America to 8.1% in Europe (9–11). Despite the well-documented risks to both mother and fetus, 32% of women who smoke daily continue to do so during pregnancy (9, 10). Despite efforts to reduce or refrain from tobacco smoking during pregnancy, up to 70% relapse after delivery (12–14).

Achieving a successful pregnancy requires the maternal immune system to adapt and tolerate a fetus that is genetically distinct. Pregnancy itself triggers a maternal inflammatory reaction, including neutrophil activation, which has been observed even in normal pregnancies. It is noteworthy that healthy pregnant women in the third trimester exhibit activation of peripheral blood leukocytes and an increase in the proportion of granulocytes compared to non-pregnant controls (15). Smoking exposure heightens the vulnerability of pregnant women by compromising immune responses, such as neutrophilia and lung inflammation (16), triggering the release of chemokines and cytokines, and causing oxidative damage (17) to lung tissue, thereby contributing to chronic lung injury (18). Recent studies have primarily focused on CS-induced lung disorders during pregnancy (19). However, research examining transcriptome changes in maternal lungs during lactation to elucidate the underlying mechanisms remains limited. Since both pregnancy and CS-exposure induce physiological changes in the lungs, it is important to understand how these changes might interact and potentially amplify each other.

It is important to note that the gut microbiome undergoes significant changes during pregnancy (20). Beyond affecting the lungs, CS can influence the gut microbiota by promoting shifts in gut microbial communities (21). Besides the effect of CS on gut function (22–24), it also influences the interconnection between the metabolic by-products of these microbes (25) and lung function, often referred to as the gut microbiome–lung axis (26, 27). Alterations in the gut microbiome resulting from the combined effects of pregnancy and CS exposure could lead to substantial downstream impacts on systemic immune responses, potentially worsening lung disorders. These alterations could contribute to the development of pregnancy-related complications, with significant consequences for maternal and infant health (19).

The gut microbiome plays a critical role in the physiological and immunological maturation and homeostasis, directly or through their metabolites such as SCFAs. SCFAs promote epithelial barrier integrity and stimulate mucus production (28). SCFAs have multiple functions in lung health (28, 29), including regulation of neutrophils (30).

This study investigates the separate and combined effects of pregnancy and CS exposure on lung transcriptomics, gut microbiota composition, and SCFA levels. By analyzing both pregnant and non-pregnant mice exposed to either CS or air, the research highlights increased sensitivity to CS during pregnancy and investigates potential mechanisms, focusing on the amplified neutrophil response and gut-lung interactions.

## 2 Materials and methods

### 2.1 Animals

Eight-week-old BALB/cByJ mice, an inbred strain (n = 90; 60 females, 30 males), were obtained in a single batch from Charles River Laboratories and housed in the same room in the animal facility of Utrecht University at controlled temperature (21 ± 2°C) and humidity (50-55%), with a 12:12 hours light/dark cycle (lights on from 7.00 am - 7.00 pm) and *ad libitum* access to pellet food (AIN-93G, Ssniff Spezialdiäten, Germany) and tap water. Upon arrival, mice were housed in groups (female: 6/cage; male: 5/cage) during acclimatization period in filter-topped makrolon (static microisolator) cages (22 cm ×16 cm ×14 cm, floor area 350 cm^2^, Tecnilab- BMI, Someren, the Netherlands); wood-chip bedding (Tecnilab- BMI, Someren, the Netherlands) and tissues (VWR, Amsterdam, the Netherlands) were available as cage enrichment at the animal facility of Utrecht University.

### 2.2 Study design

The animals included in this study were originally part of a previous *in vivo* study, where they were used to generate offspring for the required measurements and research (31). At the end of the lactation period, the offspring were separated to participate in a subsequent experiment (31). This study specifically focused on the pregnant and non-pregnant female mice. After a 2-week acclimatization period, sixty female mice were housed in pairs (2 females per cage) and randomly assigned to either the air or CS exposure groups using simple randomization. To initiate mating, one male was introduced into the home-cage of two females for a period of 4 days. After mating, the male was removed from the cage and two female mice were kept together in cages until the end of the lactation period, which lasted approximately six weeks. The first day of mating during the four-day mating period was considered as the start of pregnancy, and the pups were delivered within a three-day period (day 21-23). Females were divided into four groups, pregnant (n = 11) and non-pregnant (n = 13) mice exposed to air and pregnant (n = 17) and non-pregnant (n = 19) mice exposed to CS. All females, regardless of exposure group, were provided with a standard soy protein-based AIN-93G diet from the first day of the acclimatization period until the last day of the experiment.

### 2.3 CS exposure procedure

**Figure 1:**
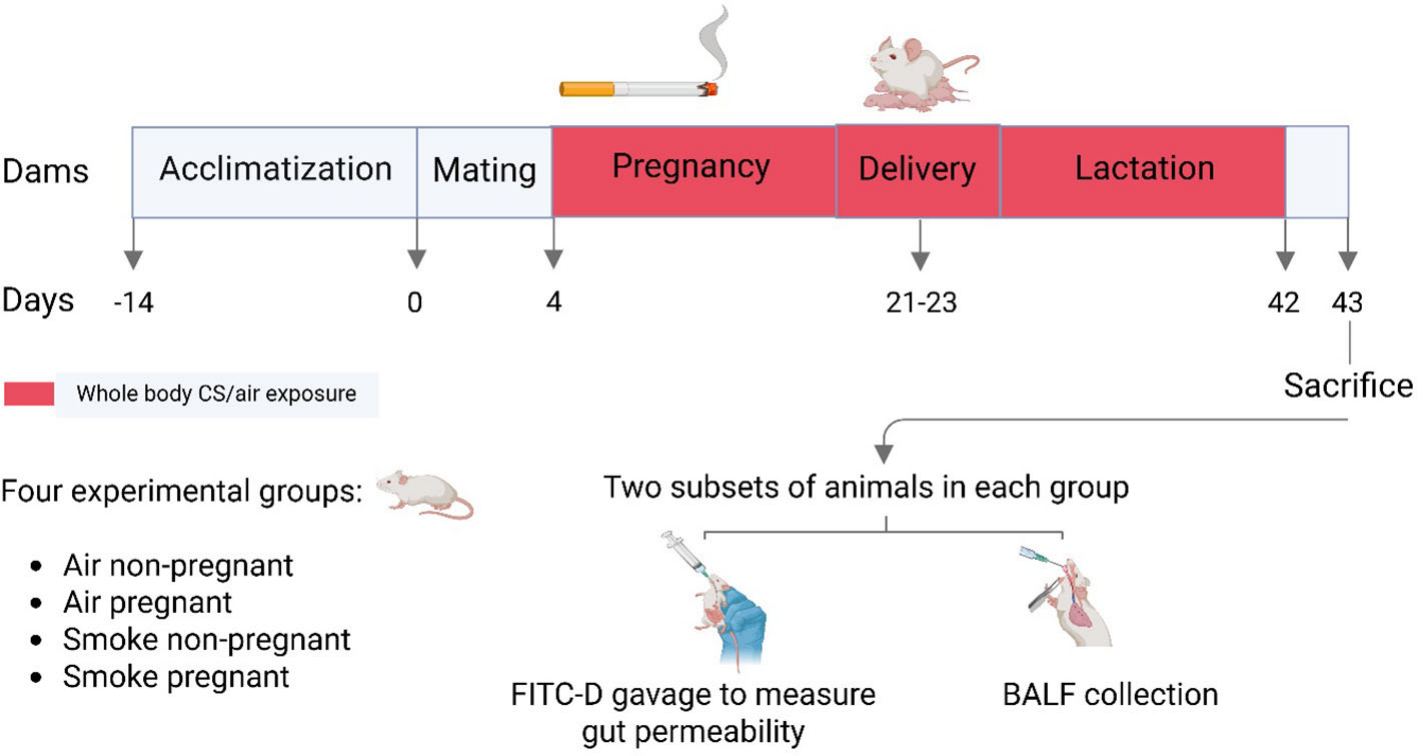
Schematic outline of the study design. After a 2-week acclimatization period, female mice were randomly assigned to either the air- or CS-exposed groups. Mating occurred by introducing one male mouse to a cage with two females for a duration of 4 days. Pups were delivered within a three-day period. Pregnant dams during pregnancy and lactation, as well as non-pregnant dams during the same period, underwent whole-body exposure to either air or diluted mainstream CS in specially designed chambers. Mice were sacrificed 24 hours after the final CS exposure, either for BALF and sample collection or for intestinal fluorescein permeability testing.

After 4 days of being in the presence of a male until the end of lactation, the pregnant and non-pregnant female mice were exposed to CS from the reference cigarettes 1R6F (University of Kentucky, Lexington, Kentucky) using a smoke apparatus as described previously (31). The smoke apparatus consists of a smoke box with four chambers (9 mice/chamber), ensuring that all animals receive equal smoke exposure. Mice were gradually acclimatized to whole body CS exposure by increasing the number of cigarettes during the first days of the experiment. Exposure started with 4 cigarettes on days 1 and 2, followed by 6 on day 3, 8 on day 4, 10 on day 5, 12 on day 6, and reached 14 cigarettes per day from day 7 onward (approximately 50 min/day). This exposure regimen was maintained 7 days/week, for 6 weeks (from pregnancy day 4 until the end of lactation). Carbon monoxide (CO) concentration and total particulate matter (TPM) were measured to monitor smoke exposure. CO concentration throughout the exposure period amounted to approximately 300–400 ppm. The mass concentration of cigarette smoke TPM was determined by gravimetric analysis using a type A/E glass fiber filter (PALL life sciences, Morelos, Mexico). The TPM concentration in the smoke exposure box likely started at around 237 μg/L and gradually increased to approximately 828 μg/L when 14 cigarettes were used for the remainder of the experiment (31). Twenty-four hours after the final CS exposure, mice (approximately 16.5 weeks old) were euthanized via intraperitoneal injection of an overdose of pentobarbital (0.15 mL per 25 g body weight; 20% solution; 200 mg/mL; Nembutal™, Ceva Santé Animale, The Netherlands) to facilitate sample collection (n = 6–10 per group), as detailed in paragraph 2.4. Additionally, a separate subset of mice (5–9 per group) underwent intestinal fluorescein permeability testing and was sacrificed using a different method, as detailed in paragraph 2.9. A schematic overview of the study design is shown in **Figure 1**. Smoking during pregnancy and lactation for 6 weeks resulted in a significantly lower spleen-to-body weight ratio in CS-exposed mice compared to air-exposed mice (**Supplementary Table 1**).

### 2.4 Collection of serum and bronchoalveolar lavage fluid

Immediately after euthanasia, blood samples were obtained from a subset of mice [air non-pregnant (n = 7), air pregnant (n = 6), smoke non-pregnant (n = 10), smoke pregnant (n = 9)] via cardiac puncture to ensure sufficient blood volume, in accordance with ethical guidelines for animal handling and welfare. The blood coagulated for 30 minutes at room temperature, centrifuged at 12,000 RPM for 10 minutes, and the serum samples were collected and stored at -20°C until further analysis.

To collect BALF, a cannula was placed into the trachea and lungs were gently lavaged *in situ* with 1 mL pre-warmed pyrogen-free saline solution (0.9% NaCl) supplemented with protease inhibitor cocktail tablets (Complete Mini, Roche Diagnostics, Mannheim, Germany) at 37°C. The lungs were lavaged three more times with 1 mL saline solution (0.9% NaCl, 37°C). Collected BALF samples were cooled on ice and centrifuged at 400 g at 4°C for 5 min. The supernatant of the first lavage was used for cytokine measurement. The cell pellets from the 4 lavages were pooled per animal, resuspended in 150 μl cold PBS and counted under light microscopy using a Bürker-Türk chamber at 100× magnification. Differential BALF cell counts were performed on air-dried cytospin preparations stained with DiffQuik™ (Merz & Dade A.G., Düdingen, Switzerland) and the numbers of total cells, macrophages, neutrophils, and lymphocytes were determined based on established morphological standards (31).

### 2.5 Analysis of T cell proliferation by flow cytometry

Fresh lungs from both subsets were collected aseptically, transferred to a sterile 6-well plate, and cut into small pieces. Each well received 1.5 mL of enzymatic digestion buffer containing DNase I and Collagenase A (Roche Diagnostics), and the tissues were incubated for 30 minutes at 37°C in a CO_2_ incubator. Digestion was stopped by adding 0.5 mL of pre-warmed fetal calf serum (FBS; Bodinco, The Netherlands) to each well. The digested tissue, including remaining fragments, was transferred onto a 70 µm nylon cell strainer placed over a 50 mL tube. The wells were rinsed several times with 1–2 mL PBS to reduce cell loss. Tissue was gently dissociated through the strainer using a 1 mL syringe plunger, and the resulting suspension was rinsed with 10 mL of plain RPMI 1640 medium (Lonza, The Netherlands). Cells were centrifuged at 1400 RPM for 5 minutes at 4°C. After discarding the supernatant, the cell pellet was resuspended in 1 mL of red blood cell lysis buffer (Thermo Scientific Chemicals, USA) and incubated on ice for 4 minutes. The lysis reaction was stopped by adding 5 mL of complete RPMI 1640 medium supplemented with 10% heat-inactivated FBS and penicillin (100 U/mL)/streptomycin (100 µg/mL; Sigma-Aldrich). Cells were washed again by centrifugation under the same conditions and finally resuspended in 2 mL of complete medium. Total cell counts were determined using a Beckman Z1 Coulter Particle Counter (Beckman, USA), and 1 × 10^6^ cells per well were used for subsequent staining and flow cytometry analysis.

To analyze T cell subsets in the lungs, the isolated cells were first blocked for 2 min at 4°C using anti-mouse CD16/CD32 antibodies (Mouse BD Fc Block; BD Pharmingen, San Jose, CA, USA) and subsequently resuspended in FACS buffer (1% bovine serum albumin (BSA) in PBS). After washing, cells were incubated at room temperature for 1 h followed by surface staining with CD4 Brilliant Violet 510, CCR6-PE (BioLegend, San Diego, CA, USA), CD69-PE-Cy7, CXCR3-PE, CD25-PerCP-Cy5.5, (eBiosciences, Thermo Fisher Scientific, San Diego, CA, USA) and T1/ST2-FITC (MD Biosciences, St. Paul, MN, USA). Viable cells were identified using a fixable viability Dye-eFluor^®^ 780 (eBioscience, San Diego, USA). For intracellular staining, cells were fixed and permeabilized using a fixation/permeabilization buffer set, according to manufacturer’s protocol (eBioscience, San Diego, USA), and then stained with Foxp3-FITC, GATA3- PerCP-eFluor710, RORγt-PE (eBiosciences, San Diego, USA), and Tbet- Alexa Fluor647 (BioLegend, San Diego, CA, USA) antibodies (31). Flow cytometric acquisition was conducted with FACS Canto II (BD Biosciences, Franklin Lakes, NJ, USA) and results were analyzed using Flowlogic Software (Inivai Technologies, Victoria, Australia). The gating strategy used to identify and analyze T cell subsets is provided in **Supplementary Figure 8**.

### 2.6 Enzyme-linked immunosorbent assa

The concentrations of S100A8/A9 (calprotectin), chemokine (C-X-C motif) ligand 1 (CXCL1) or keratinocyte-derived cytokine (KC), CXCL2 or macrophage inflammatory protein-2 (MIP2), and interleukin (IL-17A) (R&D systems, Minneapolis, USA) were determined in BALF and fecal samples using ELISA kits according to manufacturer’s instructions.

### 2.7 S100A8/A9 measurement in supernatants of ileum homogenates

The ileums of animals from both subsets were homogenized in cold RIPA buffer (Thermo Fisher, Massachusetts, USA)/phosphate-buffered saline (PBS) containing protease inhibitors (Complete Mini, EDTA-free Protease Inhibitor Cocktail, Sigma- Aldrich, Zwijndrecht, the Netherlands) using the Precellys 24 tissue homogenizer (Bertin Technologies, France). Thereafter, ileum homogenates were centrifuged (17600 g, 10 min, 4°C) and the supernatant was collected and stored at -20°C for further analyses. S100A8/A9 levels were measured by ELISA (Mouse S100A8/A9 R&D systems, Minneapolis, USA) according to the manufacturer’s instructions.

### 2.8 IgA measurement in fecal contents

Fecal samples were collected by placing each mouse in a separate clean box for 3–5 minutes one day before sacrifice. Freshly defecated fecal pellets, uncontaminated with urine, were sampled, snap-frozen in liquid nitrogen, and stored at -80°C until further analysis. The wet weight of feces samples ranged from 6 to 19 mg (median: 12 mg). Extraction buffer (PBS [pH=7.4], protease inhibitor cocktail (Sigma-Aldrich, Germany) and 0.01% sodium azide), was added to each sample at a ratio of 1 mL buffer to 100 mg fecal sample. Samples were thoroughly homogenized by a homogenizer (Bertin Technologies, France) at 6000 g for 20 s. Fecal suspensions were centrifuged at 14000 g for 10 min at 4°C and stored at -20°C until further use. Fecal IgA levels were measured in samples from both subsets, using a mouse IgA ELISA kit (Thermo Fisher Scientific, The Netherlands), following the manufacturer’s instructions. After determining the optimal sample dilution, a 1:200 dilution was used for the assay.

### 2.9 Intestinal fluorescein permeability

Twenty hours after the last CS exposure, a subset of mice (air non-pregnant (n = 6), air pregnant (n = 5), smoke non-pregnant (n = 9), and smoke pregnant (n = 8)) were fasted for 4 hours and then administered fluorescein isothiocyanate (FITC)-dextran (FD; 4 kDa, 46944, Sigma-Aldrich, Missouri, USA) by oral gavage at a dose of 600 mg/kg (32). One hour after gavage, blood was collected via retro-orbital bleeding under isoflurane anesthesia, followed by euthanasia through cervical dislocation. Anesthesia was induced by placing mice in a sealed plexiglass chamber with 4-5% isoflurane delivered in compressed air to ensure rapid onset. Serum separator clot activator tubes (Greiner Bio-One, Italy) were used for blood collection and serum was isolated after centrifugation (1500 g) and diluted 1:5 (v/v, FD) in sterile PBS. Fluorescence intensity was measured spectrophotometrically (λex 485 nm, λem 535 nm for FD) with a fluorescent microplate reader (GloMax Discover, Promega, USA) in black-wall 96-well plates. The fluorescent emission intensity was converted into fluorescein flux per hour with the help of FD standard curves.

### 2.10 Quantification of cecal short-chain fatty acids

The concentrations of SCFA, including acetic, propionic, butyric, isobutyric, valeric and isovaleric acids in the cecal samples from both subsets were quantified as described before (23, 31). Cecum contents were collected and immediately frozen on dry ice and stored at -80°C until analysis. For sample preparation, frozen samples were homogenized in ice-cold PBS at a ratio of at least four times the sample weight. Homogenization was performed thoroughly by pipetting and vortexing to ensure complete resuspension and the absence of visible clumps, which is critical for accurate SCFA extraction. Homogenates were centrifuged at 13000 RPM for 10 minutes at 4°C. The clear supernatant was collected and stored at -80°C until analysis. SCFAs were quantitatively determined using a Shimadzu GC2025 gas chromatograph (Shimadzu Corporation, Kyoto, Japan) equipped with a flame ionization detector; 2-ethylbutyric acid was used as an internal standard.

### 2.11 DNA extraction of fecal contents and library construction

Fecal samples were collected from mice in both subsets (prior to sacrifice and, when applicable, before FITC-D gavage) and stored individually at -80°C for future use. Total DNA extraction and library construction were performed by BGI Genomics (Shenzhen, China). Total bacterial DNA was extracted using MagPure Stool DNA KF Kit B (MD5115, Magen, China) following the manufacturer’s instructions. DNA was quantified with Qubit Fluorometer (Invitrogen, Carlsbad, USA) using a Qubit dsDNA BR Assay Kit (Q32850, Invitrogen, USA) and DNA quality was assessed by electrophoresis of an aliquot on 1% agarose gel.

The variable regions V3-V4 of the bacterial 16S rRNA gene were amplified using degenerate PCR primers 338F (5’-ACTCCTACGGGAGGCAGCAG-3’) and 806R (5’-GGACTACHVGGGTWTCTAAT-3’), which contain mixed nucleotide positions to target a broad range of bacterial taxa. Both forward and reverse primers were tagged with Illumina adapter, pad, and linker sequences. PCR enrichment was performed in a 50 μL reaction mixture containing 30 ng template, fusion PCR primers, and PCR master mix. PCR cycling conditions were as follows: 94°C for 3 minutes, 30 cycles of 94°C for 30 seconds, 55°C for 45 seconds, 72°C for 45 seconds, and final extension for 10 minutes at 72°C for 10 minutes. The PCR products were purified with AMPure XP beads (Beckman Coulter, Brea, CA, USA) and eluted in elution buffer. Libraries were qualified by Agilent 2100 Bioanalyzer (Agilent, CA, USA).

### 2.12 Gut microbiota sequencing and bioinformatic analysis

To determine fecal microbial diversity and composition in dams, 16S rRNA gene sequencing was performed on samples from both subsets. The validated libraries were used for sequencing by BGI (Shenzhen, China) on the DNBSEQ-G400 platform (BGI, Shenzhen, China), generating 2 × 300 bp paired-end reads with a coverage of 50k reads (clean tags per sample).

The raw reads were filtered to remove adaptors, low-quality as well as ambiguous bases, and low-complexity reads. Next, paired-end reads were added to tags by the Fast Length Adjustment of Short reads program (FLASH, version 1.2.11). Tags were clustered into Operational Taxonomic Unit (OTU) with 97% similarity using USEARCH (v7.0.1090). All tags were mapped to OTU representative sequences using USEARCH GLOBAL and results were included in an OTU abundance table. OTU representative sequences were aligned against the database for taxonomic annotation by RDP classifier (v2.2) software.

Partial least squares discrimination analysis (PLS-DA) was used as a linear classification model to predict the differences between groups. The Bray-Curtis distance serves as a widely-used index to measure dissimilarity between groups. The Analysis of Similarities (ANOSIM) is a non-parametric, rank-based method that uses distance metrics to detect dissimilarities between groups or changes in community structure. To further analyze significant differences in microbiome structure between groups, the Multi-Response Permutation Procedure (MRPP) was applied as a significance analysis method.

Gplots, mothur (v.1.31.2), and QIIME (v1.80) packages based on R (v3.1.1) were used to show relative abundance, alpha diversity (within groups), and beta diversity (between groups) results. Species abundance at the genus level was calculated after annotation. The bar plot illustrating the relative abundance of the most prevalent microbiota at the genus level was generated.

### 2.13 RNA extraction of lung tissue, library construction, RNA sequencing, and data analysis

RNA sequencing (RNA-seq) was conducted on lung tissue samples obtained from non-pregnant and pregnant mice exposed to air or CS, using animals from the BALF subset. RNA extraction from snap-frozen lung tissues was carried out using TRIzol^®^ Reagent (Invitrogen, Carlsbad, USA). The integrity and quantitation of the extracted RNA were evaluated by BGI (Shenzhen, China) using the RNA Nano 6000 Assay Kit on the Bioanalyzer 2100 system (Agilent Technologies, CA, USA), with RNA integrity number values exceeding 7. Libraries were constructed from samples of acceptable quality for strand-specific mRNA sequencing on the DNA nanoball (DNB) SEQ platform G400 (BGI, Shenzhen, China) using paired-end sequencing (PE150). The study specifically focused on the Mus musculus species, and data were retrieved from NCBI, referencing the GRCm39 version of the mouse genome.

To ensure the quality of the raw data, FASTQ-formatted raw reads underwent initial processing with SOAP nuke to obtain clean reads. Reads containing adapter, reads containing ploy-N and low-quality reads were removed from the raw data. In addition, Q20, Q30 and GC content of the clean data were calculated (80% of bases with quality score ≥ Q20). All downstream analyses were based on the high-quality clean data. Paired-end clean reads were aligned against the reference genome using Hisat2 v 2.0.5. Bowtie2 was applied to align the clean reads to the gene set. Expression level of gene was calculated by RSEM (v1.3.1).

Differential expression analysis was conducted through DESeq2 (v1.4.5), evaluating fold changes (FC), and correcting p values for multiple testing using false discovery rate (FDR) to generate adjusted p values. Genes were considered differentially expressed if FC > 1 and FDR < 0.05. Read count data were analyzed using iDEP v 0.96 (accessible at http://ge-lab.org/idep/) and the Dr. Tome system (https://biosys.bgi.com) to create principal component analysis (PCA), Gene set enrichment analysis (GSEA) and volcano plots. The expression threshold for downstream analysis was set at a minimum of 0.5 count per million (CPM) in at least two samples to remove low abundance genes, and counts data were transformed using EdgeR:28 log2(CPM + c), where constant “c” = 4. The heatmap illustrating gene expression differences across various samples was generated using heatmap (v1.0.8).

On average, each sample produced approximately 6.67 gigabases of sequencing data, resulting in an average mapping ratio of 95.79% to the reference genome and a 74.58% mapping ratio to known genes. This approach identified a total of 18,655 genes.

### 2.14 Statistical analysis

Statistical analysis was performed using GraphPad Prism v9.3.1 software (San Diego, USA) or specific functions of R packages (for microbiome composition and diversity calculation). Results are expressed as mean ± standard error of mean (SEM). Data were cleaned by identifying and excluding outliers using the Grubbs’ test. The remaining data were analyzed using a two-way ANOVA followed by Bonferroni’s *post hoc* multiple comparisons test, as specified in each figure legend. When comparing two means, Student’s t-test was performed. Differences were considered statistically significant when p < 0.05.

## 3 Results

### 3.1 Pregnancy as well as CS exposure induces lung transcriptional changes

The transcriptomes of lung tissue from four distinct groups of pregnant/non-pregnant mice exposed to air or CS were analyzed to comprehensively characterize the response to CS during pregnancy and lactation. A total of 18,655 genes met the expression threshold of 0.5 CPM in at least two samples. Among these expressed genes, 532 genes (3.49%) were dysregulated (|log2(fold-change)| > 1, FDR < 0.05) in all four comparisons. The gene symbols and IDs of all 532 differentially expressed genes (DEGs), along with their fold change (FC) and adjusted p-value based on the comparisons, are provided in **Supplementary Table 2**. Our primary objective was to explore the baseline transcriptome of air-exposed pregnant and non-pregnant mice and compare it to that of CS-exposed mice. This approach allowed us to identify differences in the transcriptomic data between pregnant and non-pregnant mice exposed to CS.

**Figure 2:**
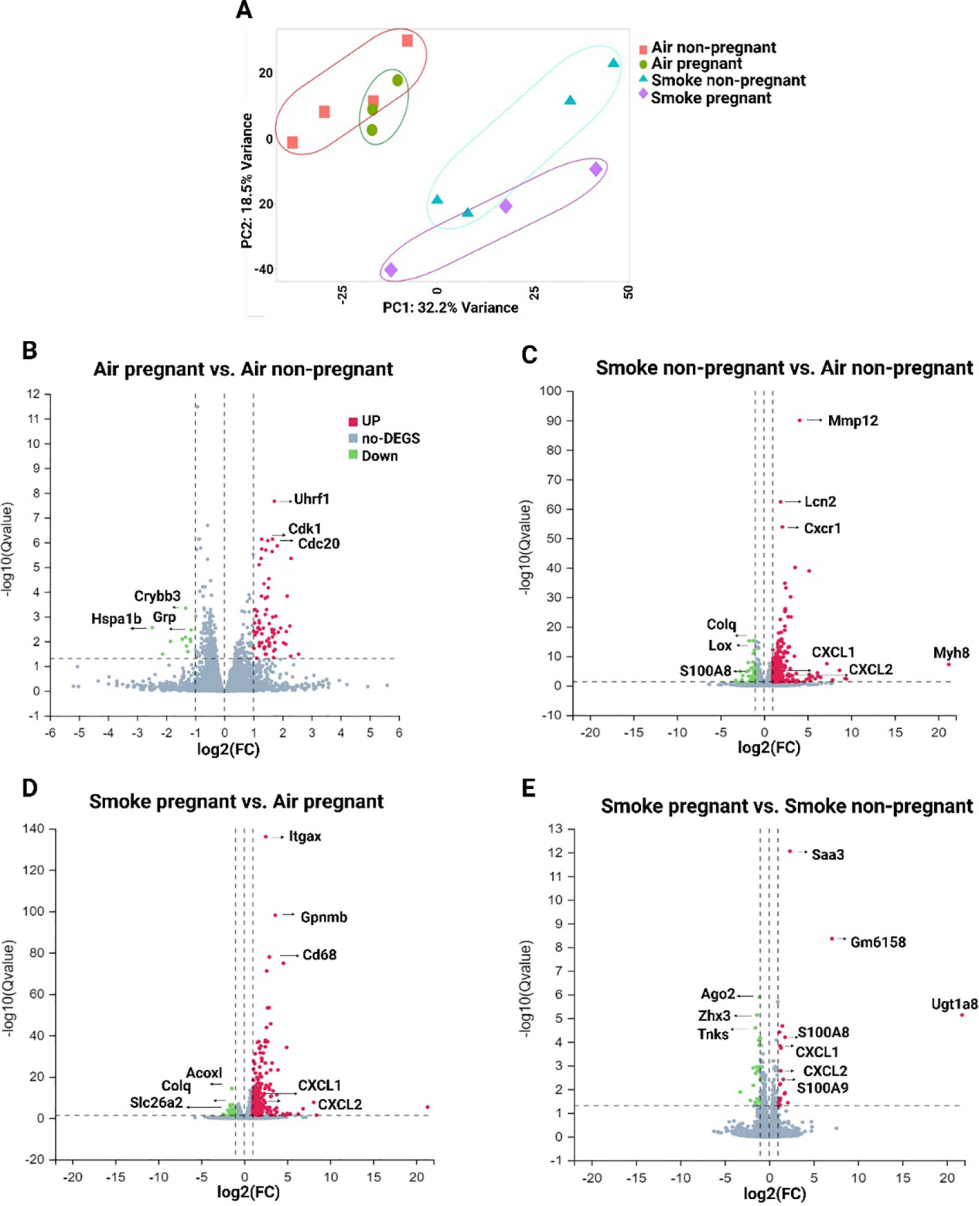
Gene expression analysis by RNA-seq in lung tissues from non-pregnant and pregnant/lactating mice exposed to CS or air. **(A)** PCA showing overall similarity and variation in the global patterns of gene expression in all four experimental groups. Volcano plots were generated to visualize the distribution of DEGs in lung tissue between different comparisons: **(B)** air pregnant vs air non-pregnant, **(C)** smoke non-pregnant vs air non-pregnant, **(D)** smoke pregnant vs air pregnant, and **(E)** smoke pregnant vs smoke non-pregnant. The X-axis of the volcano plot indicates the fold change, whereas the Y-axis shows the q-values (log-scaled). Genes above thresholds are highlighted by coloring. Each dot represents a different gene, and the red/green color of the dots categorizes the up-regulated/down-regulated genes (padj < 0.05, |log2(fold-change)| > 1). n = 3–4 mice per group (BALF subset).

To comprehend the clusters and variance in the dataset, and to simplify the high-dimensional data, PCA across all experimental groups were conducted. The results of PCA revealed a clear separation between the air- and CS-exposed groups (**Figure 2A**). The first two principal components explained 32.2% and 18.5% of the variance in gene expression. The X-axis exhibited distinct clustering of CS-exposed groups separate from air-exposed mice (for both pregnant and non-pregnant groups), while the Y-axis displayed some level of separation between the non-pregnant and pregnant CS-exposed mice (**Figure 2A**).

To provide further insight into the transcriptional profile based on the comparisons, the number of up- and downregulated genes for each comparison is illustrated by volcano plots (**Figures 2B-E**). The comparisons include air pregnant vs. air non-pregnant (72 upregulated, 11 downregulated genes) (**Figure 2B**), smoke non-pregnant vs. air non-pregnant (302 up, 47 down) (**Figure 2C**), smoke pregnant vs. air pregnant (254 up, 38 down) (**Figure 2D**), and smoke pregnant vs. smoke non-pregnant (20 up, 22 down) (**Figure 2E**). These data indicate that CS exposure, in both pregnant and non-pregnant mice, elicits a robust transcriptional response compared to air-exposed controls, with the largest number of differentially expressed genes observed in smoke-exposed non-pregnant mice (**Figure 2C**). In air-exposed mice, cell cycle–related genes such as Uhrf1 and Cdk1 were upregulated in the pregnant group compared to non-pregnant controls, possibly reflecting increased proliferative or regenerative processes during pregnancy (**Figure 2B**). Smoke exposure induced strong upregulation of inflammatory genes, notably, S100A8, S100A9, CXCL1, and CXCL2, which are associated with innate immune activation. These genes were highly upregulated in both smoke-exposed pregnant and smoke-exposed non-pregnant mice. This suggests that pregnancy does not dampen the inflammatory transcriptional response to CS, but may modulate its magnitude or downstream consequences. Together, these findings suggest that both pregnancy and CS exposure distinctly influence the pulmonary transcriptome, with CS triggering prominent inflammatory pathways and pregnancy modulating gene programs related to cell proliferation.

### 3.2 Pregnancy as well as CS exposure leads to differences in GO enrichment analysis

**Figure 3:**
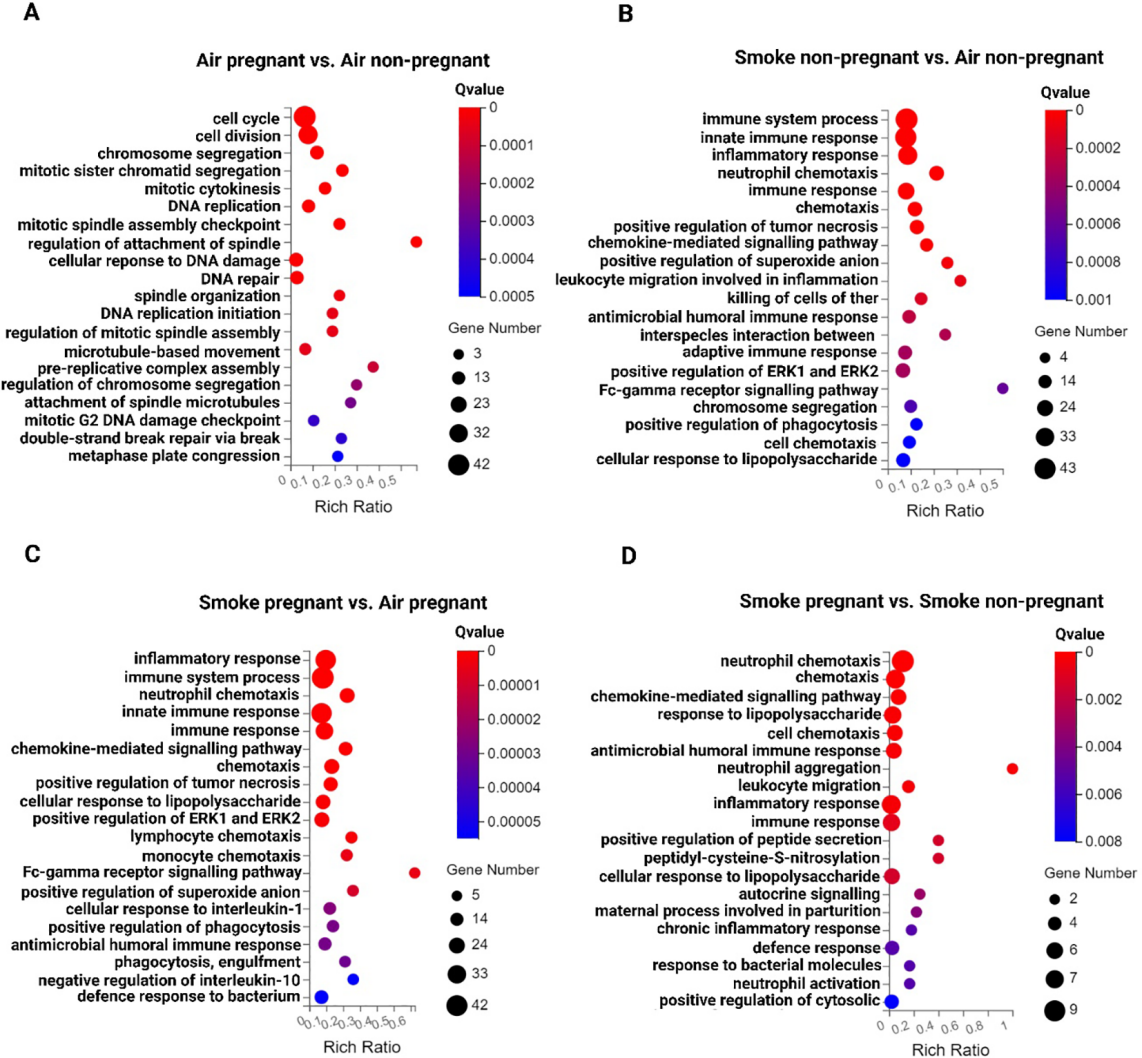
Inflammatory response pathways in the lung tissues from non-pregnant and pregnant/lactating mice exposed to CS or air. The top 20 signaling pathways from the GO enrichment analysis of DEGs across various comparisons: **(A)** air pregnant vs air non-pregnant, **(B)** smoke non-pregnant vs air non-pregnant, **(C)** smoke pregnant vs air pregnant, and **(D)** smoke pregnant vs smoke non-pregnant. Circles represent the number of genes, and colors indicate the richness factors. The color saturation corresponds to the significance of enrichment (Qvalues). n = 3–4 mice per group (BALF subset).

To identify key signaling pathways in lung tissues upon pregnancy and CS exposure, a GO enrichment analysis was performed. The top 20 significantly dysregulated pathways (padj < 0.05, |log2 (Fold Change)| > 1) are shown in **Figures 3A–D**. The analysis revealed that the top 5 biological process pathways in the lungs of pregnant air-exposed mice compared to non-pregnant mice include cell cycle, cell division, chromosome segregation, mitotic sister chromatid segregation, and mitotic cytokinesis, with no enrichment in immune response pathways (**Figure 3A**). In contrast, the top 5 pathways associated with CS exposure, compared to air-exposed mice, consistently highlighted immune-related processes, although in varying orders. These pathways included the immune system process, innate immune response, inflammatory response, neutrophil chemotaxis, and immune response (**Figures 3B, C**). Finally, the top 5 enriched pathways in CS-exposed pregnant mice compared to CS-exposed non-pregnant mice featured neutrophil chemotaxis, chemotaxis, chemokine-mediated signaling pathway, response to lipopolysaccharide, and cell chemotaxis (**Figure 3D**). This comparison emphasizes that neutrophil chemotaxis is the dominant immune pathway driving inflammation in smoke-exposed pregnant mice (**Figures 3B-D**).

### 3.3 Neutrophil chemotaxis pathway is enhanced following CS exposure in pregnant dams compared to non-pregnant mice

**Figure 4:**
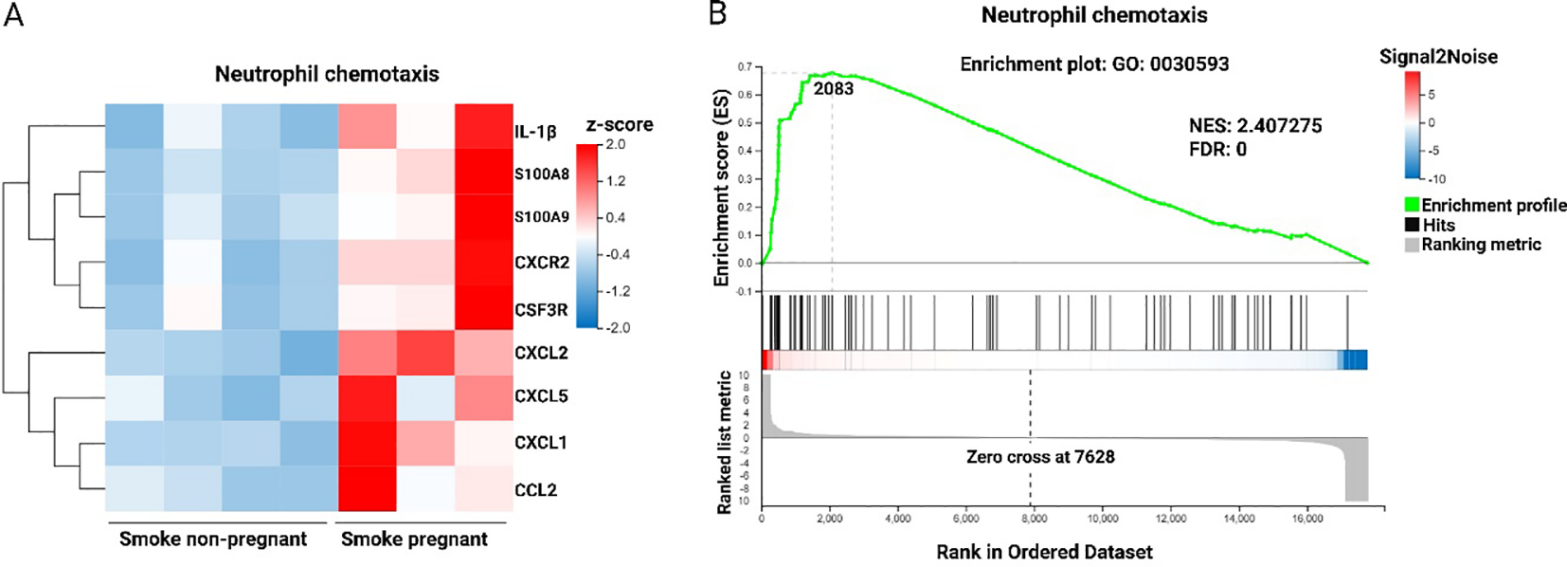
Neutrophil chemotaxis pathway is enhanced following CS exposure in pregnant dams compared to non-pregnant mice. **(A)** Heatmap of RNA-Seq expression z-scores computed for DEGs (padj < 0.05, |log2(fold-change)| > 1) in neutrophil chemotaxis pathway based on the GO enrichment analysis from the comparison of Smoke pregnant to Smoke non-pregnant. Each column in the heatmap is an individual sample. Red indicates up-regulation, and blue indicates down-regulation. **(B)** GSEA showing the enrichment of the neutrophil chemotaxis signature in the RNA-Seq profile from the comparison of pregnant compared to non-pregnant mice exposed to CS. NES, normalized enrichment score. FDR, false discovery rate. n = 3–4 mice per group (BALF subset).

To examine the influence of pregnancy on transcriptomic changes in the lungs of mice exposed to CS, transcriptomic profiles of pregnant mice were compared to those of non-pregnant mice (**Figure 3D**). The analysis revealed that neutrophil chemotaxis was the predominant pathway activated in CS-exposed pregnant dams relative to CS-exposed non-pregnant mice, as shown in **Figure 2D**. Specific genes within this pathway, including IL-1β, S100A8, S100A9, CXCR2, CSF3R, CXCL2, CXCL5, CXCL1, and CCL2, were significantly upregulated in CS-exposed pregnant mice (**Figure 4A**). In other words, pregnancy exacerbates the expression of these genes in mice exposed to CS. GSEA was conducted on the list of gene signatures to identify pathways affected by pregnancy in the two groups exposed to CS. The neutrophil chemotaxis signature was enriched in pregnant CS-exposed mice compared to non-pregnant CS-exposed mice (FDR = 0.00, NES = 2.40) (**Figure 4B**).

Additional data highlighting the impact of CS exposure on pregnancy is presented in **Supplementary Figure 1A**, which shows a heatmap of all upregulated and downregulated DEGs from the comparison of CS-exposed pregnant and non-pregnant groups. Furthermore, **Supplementary Figure 1B** provides more details on the neutrophil chemotaxis pathway by illustrating the top five enriched molecular function pathways in CS-exposed mice. These include chemokine activity, CXCR chemokine receptor binding, cytokine activity, Toll-like receptor 4 binding, and antioxidant activity.

### 3.4 Greater increase in neutrophil counts in BALF following CS exposure in pregnant dams compared to non-pregnant mice

**Figure 5:**
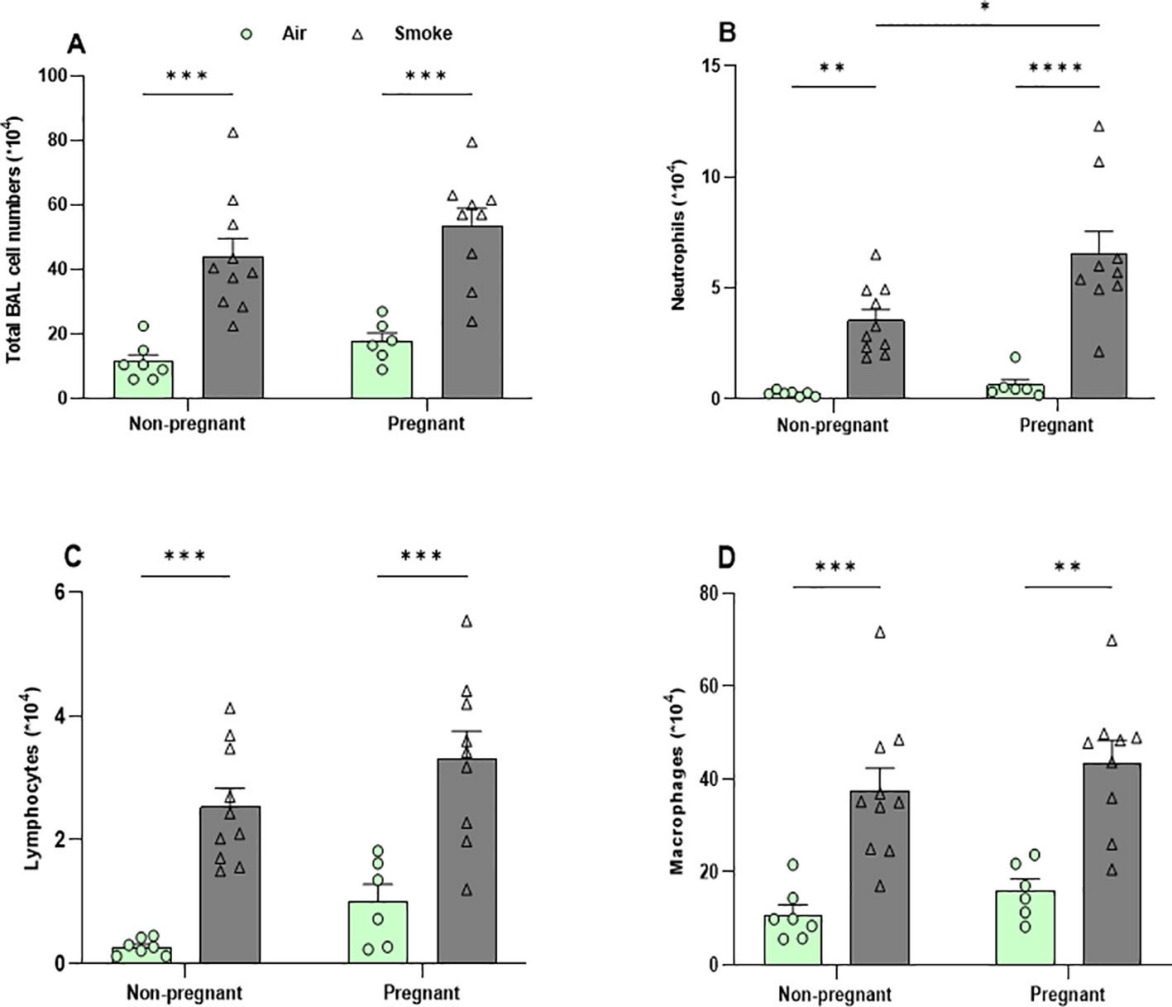
Greater increase in neutrophil counts in BALF following CS exposure in pregnant dams compared to non-pregnant mice. Lungs were lavaged, and BALF was collected for total **(A)** and differential inflammatory cell counts, including **(B)** neutrophils, **(C)** lymphocytes, and **(D)** macrophages. Data are presented as mean ± SEM; n = 6–10 mice/group (BALF subset). *p < 0.05, **p < 0.01, ***p < 0.001, and ****p < 0.0001, as determined by two-way ANOVA followed by Bonferroni’s multiple comparisons test.

Pregnancy did not influence inflammatory cells counts in the BALF of air-exposed mice. However, CS-exposed pregnant and non-pregnant mice exhibited significantly higher levels of total inflammatory cells, eosinophils, neutrophils, lymphocytes, and macrophages in the BALF compared to air-exposed mice (**Figures 5A–D**). Although both pregnant and non-pregnant dams showed an increase in neutrophil infiltration after CS exposure, the neutrophil influx was significantly higher in CS-exposed pregnant dams compared to CS-exposed non-pregnant mice, consistent with RNA-seq data showing neutrophil chemotaxis as the main contributor to the lung transcriptional response in pregnant CS-exposed mice (**Figure 5B**). **Supplementary Figures 2A–D** shows representative images of BALF cells from different experimental groups, prepared using cytospin and stained with Diff-Quik, illustrating differences in BALF cell composition, particularly variations in neutrophil abundance among groups.

### 3.5 Pregnant dams exhibit greater secretion of neutrophil chemotaxis mediators in BALF following CS exposure

**Figure 6:**
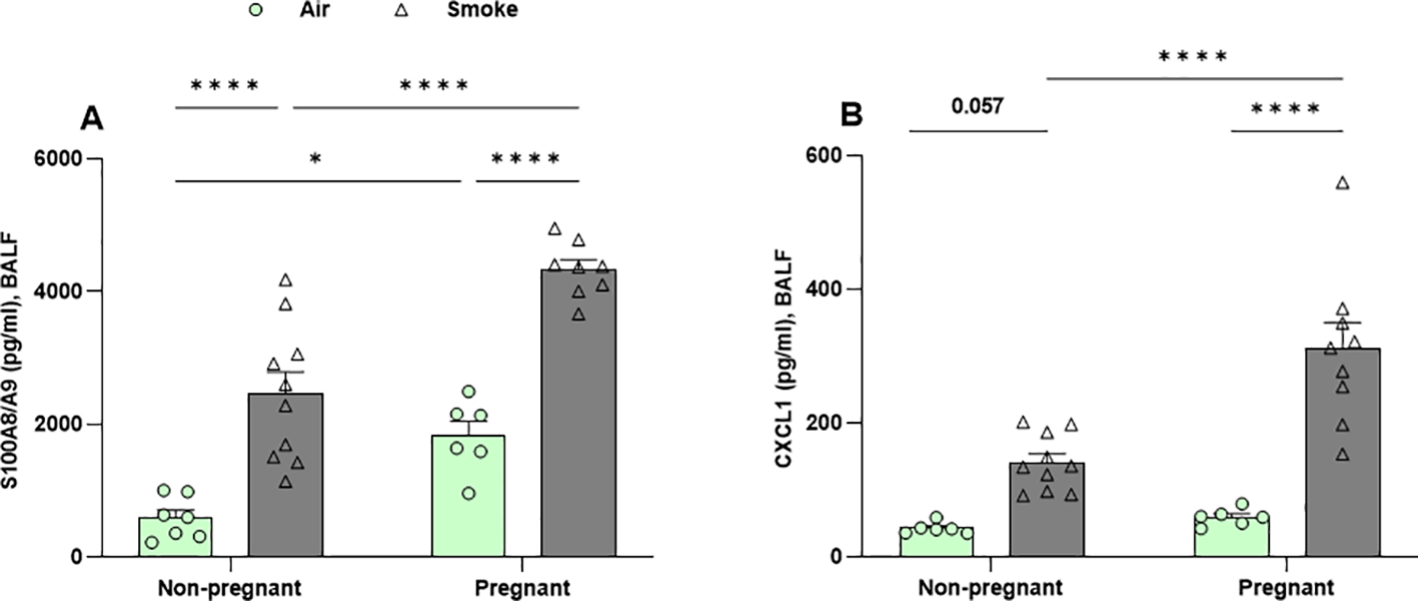
Pregnant dams exhibit greater secretion of neutrophil chemotaxis mediators in BALF following CS exposure. Concentrations of (A) S100A8/A9, and (B) CXCL1 were measured in BALF. Data are presented as mean ± SEM; n = 6-10 mice/group (BALF subset). *p < 0.05 and ****p < 0.0001, as determined by two-way ANOVA followed by Bonferroni’s multiple comparisons test.

BALF was analyzed to determine whether CS exposure increased local concentrations of neutrophil chemotaxis-associated mediators, S100A8/A9 and CXCL1, in pregnant dams compared to non-pregnant dams. Pregnant mice exposed to air exhibited a significant increase in S100A8/A9 (calprotectin) concentrations compared to non-pregnant mice (**Figure 5A**). No differences were observed in CXCL1 concentrations between the air-exposed groups (**Figure 5B**). CS-exposed mice had significantly higher BALF levels of S100A8/A9 and CXCL1 compared to air-exposed mice in both pregnant and non-pregnant mice, except for CXCL1, which showed a trend in non-pregnant CS-exposed mice compared to non-pregnant air-exposed group (p = 0.057) (**Figures 6A, B**).

Interestingly, CS exposure induced significantly higher S100A8/A9 and CXCL1 levels in pregnant mice compared to non-pregnant mice. These mediators showed a similar trend as the neutrophil counts in the BALF (**Figure 5B**) and aligned with RNA-seq data highlighting neutrophil chemotaxis as the key lung response in pregnant CS-exposed mice, as evidenced by related gene expression changes in the volcano plots (**Figures 2C–E**).

Additionally, no significant effect of pregnancy or CS exposure was observed on CXCL2 concentrations in BALF (**Supplementary Figure 3D**).

### 3.6 CS exposure increased the frequency of Th17 cells in the lungs of pregnant mice

**Figure 7:**
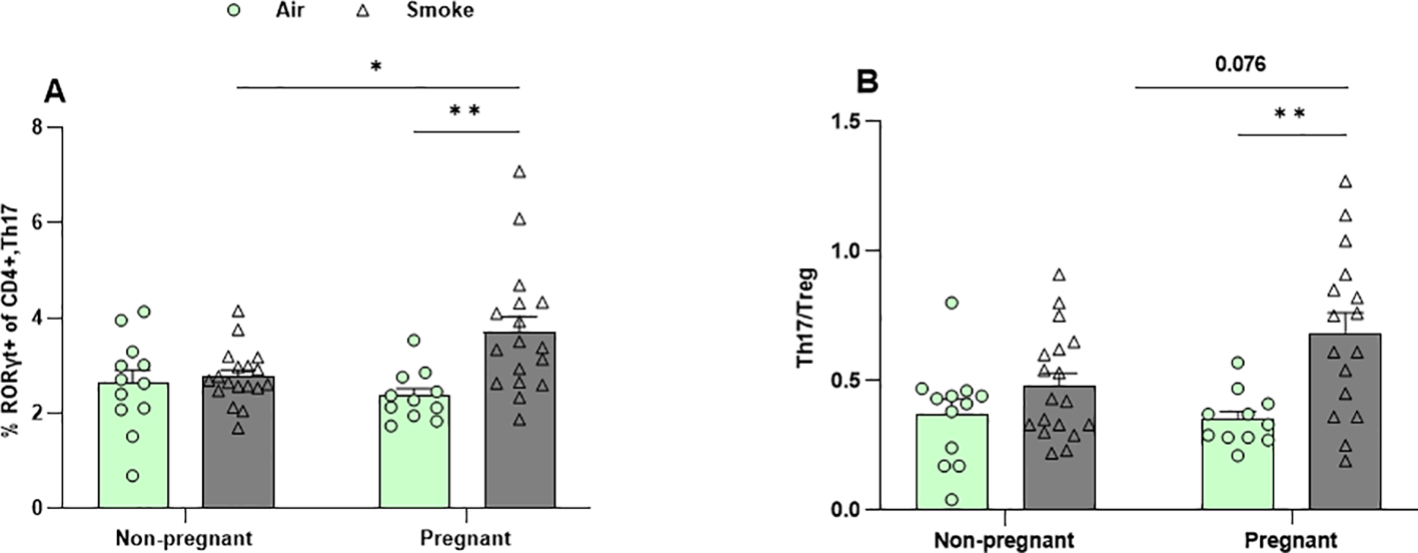
CS exposure increased the frequency of Th17 cells in the lungs of pregnant mice. Lung cell suspensions were analyzed for the percentage of **(A)** Th17 cells (RORγt+ CD4+ cells) and **(B)** Th17/Treg ratio. Data are presented as mean ± SEM; n = 11–19 mice/group from both subsets. *p < 0.05, and **p < 0.01, as determined by two-way ANOVA followed by Bonferroni’s multiple comparisons test.

Flow cytometric analysis of lung cell suspensions was conducted to measure the presence of different T-cell subsets (**Figures 7A, B**; **Supplementary Figures 3A–C**). The frequency of Th2 cells (GATA3+, T1ST2+ CD4+ cells), Th1 cells (Tbet+ CXCR3+ CD4+ cells), and Treg cells (CD25+, Foxp3+ CD4+ cells) remained unchanged in both CS- and air-exposed groups (**Supplementary Figures 3A–C**). No significant effect of pregnancy was observed on the percentage of Th17 in air-exposed groups (**Figure 7A**), and Th17/Treg ratio reflected a similar pattern (**Figure 7B**). CS exposure did not alter Th17 cell levels in non-pregnant mice. However, Th17 cells were significantly elevated in pregnant mice exposed to CS compared to both non-pregnant mice exposed to CS and pregnant mice exposed to air (**Figure 7A**). Similarly, the Th17/Treg ratio was elevated in pregnant mice exposed to CS compared to those exposed to air. However, IL-17A levels—the Th17-associated cytokine—in BALF were not affected by either pregnancy or CS exposure (**Supplementary Figure 3E**).

### 3.7 CS exposure exacerbates pregnancy-induced microbial diversity changes

**Figure 8:**
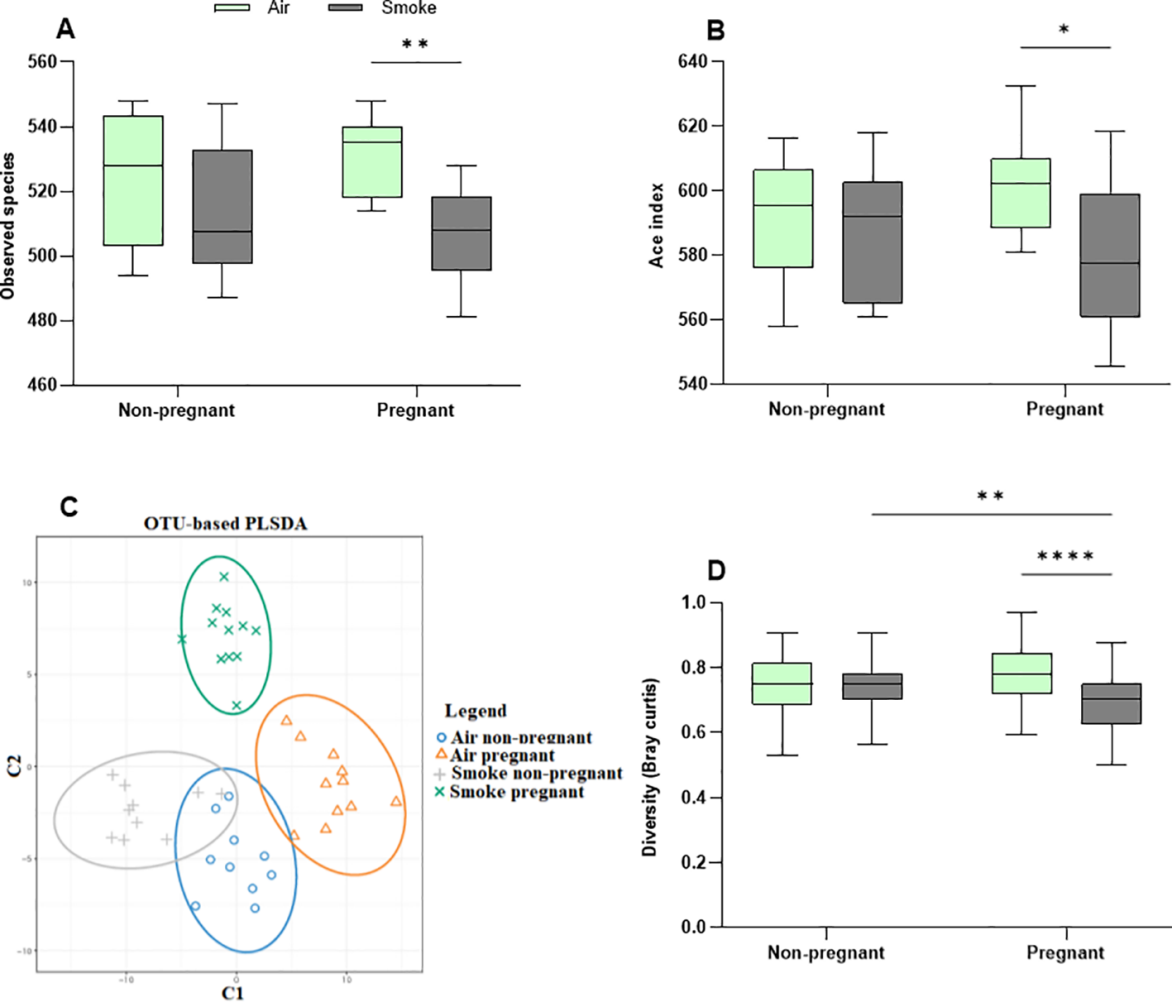
CS exposure exacerbates pregnancy-induced microbial diversity changes. Alpha diversity is depicted as **(A)** observed species and **(B)** Ace index. Beta diversity is depicted as **(C)** PLSDA and **(D)** Bray–Curtis distance index. Each point in the PLSDA plot represents a single sample and the closed areas reflect confidence ellipses. The closer the spatial distance of the samples, the more similar the species composition of the samples **(C)**. The Bray-Curtis distance serves as a widely-used index to measure dissimilarity between two communities, with values ranging from 0 to 1; a value of zero represents an exact match in community structure **(D)**. Data are presented as mean ± SEM; *n* = 10–12 mice/group from both subsets. *p < 0.05, **p < 0.01, and ****p < 0.0001, as determined by two-way ANOVA followed by Bonferroni’s multiple comparisons test.

Community richness (observed species and Ace index) (**Figures 8A, B**) and diversity (Simpson and Shannon) (**Supplementary Figures 4A, B**) were assessed to quantify alpha diversity. Pregnancy alone did not impact alpha diversity, nor did CS exposure in non-pregnant groups. However, CS exposure significantly decreased microbial richness in pregnant mice compared to air-exposed pregnant mice, reflected by reduced observed species and ACE index values. This indicates a notable decline in gut microbiota diversity in CS-exposed pregnant mice (**Figures 8A, B**). Additionally, the Simpson and Shannon indexes showed no differences among all groups, suggesting that neither pregnancy nor CS exposure affects community diversity (**Supplementary Figures 4A, B**).

Beta diversity compares the (dis-)similarities of the microbiome between different communities and was determined by PLS-DA, the Bray-Curtis distance index, MRPP, and ANOSIM. The PLS-DA plot showed apparent clusters of the pregnant groups, which were clearly distinct from the non-pregnant groups (**Figure 8C**). The results point to a distinctive effect of pregnancy on microbiota diversity, while CS exposure further contributes to the differences observed in groups. The confidence ellipses indicated overlap or more similarity in non-pregnant mice, whereas (dis-)similarity was pronounced in the confidence ellipses of pregnant groups, with distinct ellipses (**Figure 8C**).

Based on the Bray-Curtis distance index (**Figure 8D**), pregnancy did not impact gut microbiota similarity in air-exposed mice. However, the pregnant CS-exposed group exhibited significantly less dissimilarity compared to the pregnant air-exposed group, indicating greater variability in gut microbiota among pregnant mice exposed to air (**Figure 8D**). MRPP analysis revealed that pregnancy did not significantly affect the air-exposed groups. In contrast, CS exposure in non-pregnant groups approached significance (p = 0.055), while in pregnant groups, it reached significance (p = 0.04). The combined influence of pregnancy and CS exposure yielded a significant difference (p = 0.012). Taken together, the combination of pregnancy and CS exposure alters gut beta diversity in mice, as confirmed by the MRPP (p = 0.001) and ANOSIM (R = 0.126, p = 0,002) (**Supplementary Figure 4C**). CS exposure reduced beta diversity in pregnant dams compared to air-exposed mice (**Figures 8C, D**).

Gut microbiota composition did not show considerable variation among the different groups (**Supplementary Figure 5A**). The LEfSe further identified the features that most likely explain differences among the air- and CS-exposed mice (**Supplementary Figures 5B, C**). In non-pregnant mice exposed to air, *Akkermansia*, *Weissella*, *Leuconostocaceae*, and *Olsenella* were enriched, while *Cyanobacteria* was the most enriched microbe in pregnant dams exposed to air. Additionally, *Eubacteriaceae* and *Turicibacter* were among the enriched microbes in non-pregnant and pregnant dams exposed to CS, respectively (**Supplementary Figures 5B, C**). Measures of intestinal inflammation and function were analyzed to assess the potential effects of pregnancy and/or CS exposure. No significant changes were observed in ileal expression of calprotectin or in fecal IgA concentrations (**Supplementary Figures 6A–D**).

### 3.8 Pregnancy-induced increases in SCFA production were counteracted by CS exposure

**Figure 9:**
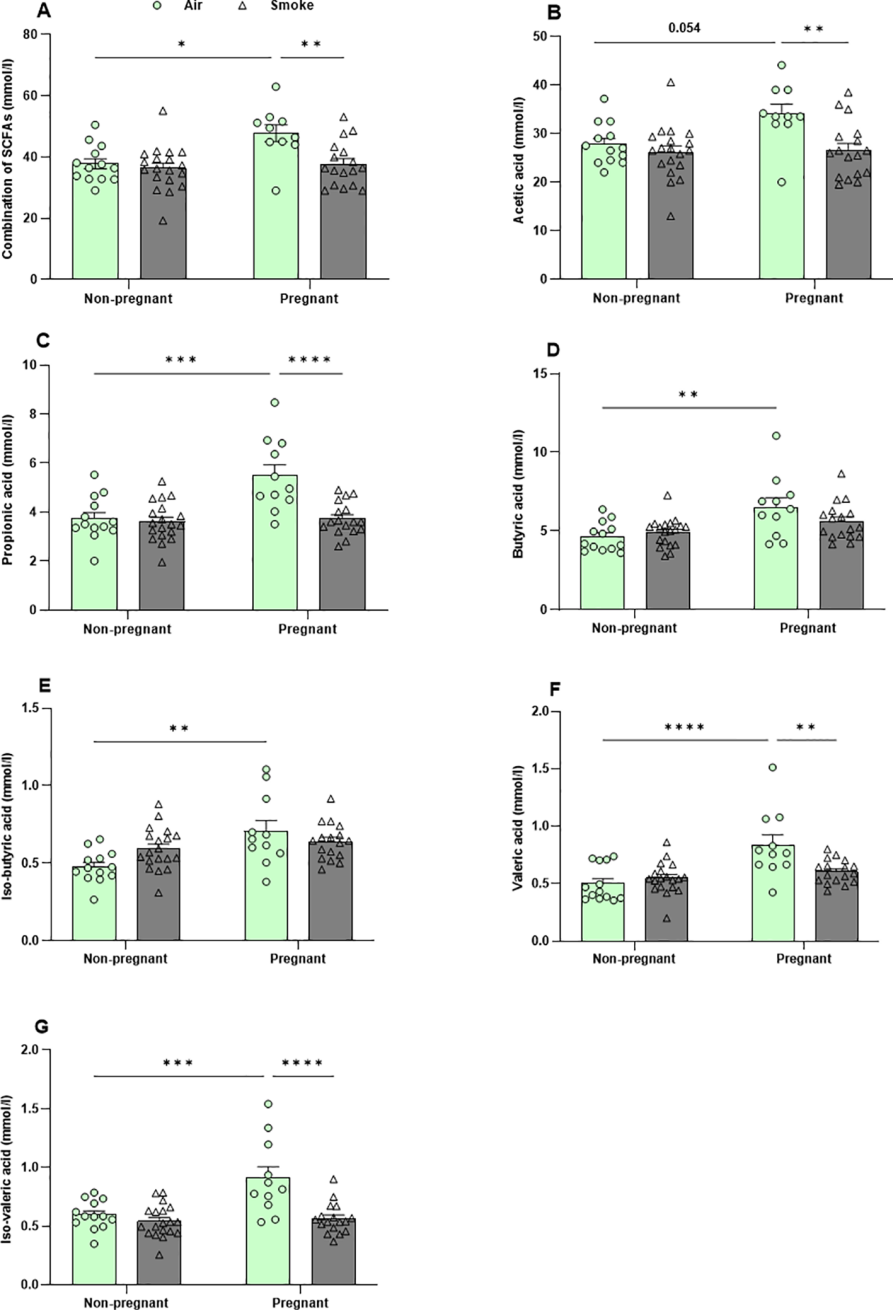
Pregnancy-induced increases in SCFA production were counteracted by CS exposure. The concentrations of **(A)** total SCFA and individual SCFA, including **(B)** acetic acid, **(C)** propionic acid, **(D)** butyric acid, **(E)** iso-butyric acid, **(F)** valeric acid, and **(G)** iso-valeric acid were measured in cecal contents. Data are presented as mean ± SEM; n = 11–19 mice/group from both subsets. *p < 0.05, **p < 0.01, ***p < 0.001, and ****p < 0.0001, as determined by two-way ANOVA followed by Bonferroni’s multiple comparisons test.

Following approximately 6 weeks of CS exposure, the cecal contents were collected and the concentrations of the six most abundant SCFA (acetate, propionate, butyrate, isobutyrate, valerate, and isovalerate) were determined. Pregnancy in air-exposed groups resulted in a significant increase in the total and individual SCFA, including acetic acid (p = 0.054), propionic acid, butyric acid, iso-butyric acid, valeric acid, and iso-valeric acid compared to non-pregnant mice, while no significant difference in the production of the SCFA could be detected in CS-exposed non-pregnant mice compared to CS-exposed pregnant mice (**Figures 9A–G**). CS exposure reduced the production of cecal SCFA (acetic acid, propionic acid, valeric acid, and iso-valeric acid) in pregnant mice compared to pregnant mice exposed to air (**Figures 9A–G**), while CS exposure did not significantly affect cecal SCFA levels in non-pregnant mice. In addition, no significant differences were found in total SCFA concentration nor the different SCFA species in serum of mice exposed to CS or air (**Supplementary Figures 7A–E**).

## 4 Discussion

Exposure to cigarette smoke, whether through passive smoking, primarily as indoor air pollution from environmental smoke, or through active smoking, is the leading cause of preventable diseases and deaths worldwide (3, 5, 6). Despite the significant health risks associated with smoking during pregnancy for both mothers and infants, over 30% of women worldwide are exposed to environmental tobacco smoke (3). Additionally, 32% of women who smoke daily, continue to do so during pregnancy around the world (9). During pregnancy, smoking increases the risk of complications such as miscarriage, stillbirth, preterm birth, fetal growth restriction, and congenital disorders (2, 33). Exposure to CS also raises the risk of respiratory diseases, as there is no safe level of exposure (2, 5).

We and others showed that CS exposure, as a proxy for both environmental tobacco smoke and air pollution, induces alterations in maternal immunity (16, 25). Moreover, there is substantial evidence indicating that both pregnancy and smoking can independently affect the diversity and composition of gut microbiota (20–22, 34). The microbiome and changes in the microbiome may also alter the clinical outcomes of smoking (26).

The current study is the first to simultaneously measure lung transcriptomics, gut microbiota composition and associated metabolites in pregnant dams exposed to CS.

The transcriptomic profiles of lung tissues from dams showed that pregnancy in air-exposed mice primarily affected genes related to cell division and mitosis, with no significant impact on immune system-related genes. In contrast, CS exposure in both pregnant and non-pregnant groups primarily influenced immune response pathways, including innate immune responses, inflammatory responses. Pregnancy amplifies the inflammatory response to CS exposure, particularly by enhancing neutrophil infiltration in the lungs. Notably, neutrophil chemotaxis was the most enriched pathway, with nine upregulated genes. These pro-inflammatory genes, including IL-1β, S100A8, S100A9, CXCR2, CSF3R, CXCL2, CXCL5, CXCL1, and CCL2, showed increased expression in CS-exposed pregnant mice. These findings strongly suggest that neutrophils are the primary drivers of the immune response in CS-exposed pregnant dams. To substantiate this, the various cell types present in the BALF were differentiated and analyzed. Pregnancy alone in air-exposed mice did not significantly affect the number of inflammatory cells in BALF. However, both non-pregnant and pregnant mice exposed to CS exhibited a significant increase in the number of inflammatory cells in BALF, including neutrophils, lymphocytes, and macrophages compared to air-exposed controls. Interestingly, pregnant dams exposed to CS displayed a more substantial increase in neutrophil infiltration compared to non-pregnant mice exposed to CS, as previously demonstrated by our group (16). These findings are consistent with and support the RNA-seq results. Studies in mice indicate that neutrophils infiltrating the airways not only contribute to initiating airway inflammation but also prolong the inflammatory response (35).

Moreover, the levels of neutrophil chemotaxis-associated mediators, S100A8 and S100A9, were elevated in the BALF of pregnant mice in the air-exposed group. CS exposure further increased the levels of S100A8, S100A9, and CXCL1 in both pregnant and non-pregnant groups. The combined effect of pregnancy and CS exposure closely mirrored the pattern of neutrophil infiltration and further validated the RNA-seq data, showing significantly higher S100A8, S100A9, and CXCL1 levels in CS-exposed pregnant dams compared to CS-exposed non-pregnant mice. Neutrophils, as the first responders to acute inflammation, migrate from the circulatory system into inflamed lung tissue by following a chemokine gradient, such as CXCL1 (36). A clinical study demonstrated that S100A9 levels were higher in sputum from patients with severe asthma and neutrophil-dominant inflammation compared to sputum from eosinophil-dominant groups. Furthermore, S100A9 levels showed a significant correlation with the percentage of neutrophils in the sputum of asthma patients (37). S100A9 expression was increased in the lungs of mice, particularly in inflammatory cells and the fibrotic interstitium, in a bleomycin-induced pulmonary fibrosis model. Treatment with an S100A9 inhibitor reduced neutrophil counts in BALF in this model. In patients with idiopathic pulmonary fibrosis, BALF S100A9 levels were positively correlated with both the number and percentage of neutrophils. Furthermore, higher S100A9 levels in both serum and BALF were independently associated with poorer prognosis in IPF patients (38, 39). There are insufficient studies to explain the role of calprotectin during pregnancy. However, elevated S100A9 levels have been observed in the plasma and placentas of patients with preeclampsia, a pregnancy-specific syndrome. Furthermore, administration of S100A9 significantly increased neutrophil accumulation in the placentas of pregnant mice (40).

Neutrophils and T cells are known to mutually influence each other’s effector functions through mechanism such as chemokine and cytokine production, neutrophil extracellular trap release, and contact-dependent mechanisms (41). While pregnancy alone did not affect Th17 cell levels, we observed that CS exposure significantly increased Th17 cells in the lungs of pregnant dams compared to non-pregnant mice, mirroring the pattern seen with neutrophils. Consistent with previous studies, CS stimulation may promote Th17 cell differentiation and development through RORγt, which in turn recruit neutrophils to sites of inflammation (42). Neutrophils modulate various CD4+ subsets, including Th17 and Tregs, either enhancing or suppressing their activity (43). Th17 cells directly stimulate neutrophil activity through the release of IL-8, the human homologues of CXCL1 and CXCL2 (41). The Th17 effector cytokine IL-17 acts indirectly by stimulating epithelial cells to produce neutrophil chemoattractants such as IL-8, CXCL1, and GM-CSF (44). However, IL-17A levels in BALF were not affected by either pregnancy or CS exposure, suggesting that Th17-mediated neutrophil modulation may occur through IL-17–independent mechanisms, possibly via the release of GM-CSF, TNF-α, and IFN-γ (45).

The intricate cross-talk between the host immune system, gut microbiota composition, and their metabolites plays a crucial role in maintaining host homeostasis. This dynamic interaction shapes immune responses, influencing both local and systemic immunity (24, 25, 27).

The gut microbiota undergoes significant alterations during pregnancy, driven by hormonal changes and shifts in immune metabolism (20). However, the impact of pregnancy on gut microbiota diversity varies across studies, with no clear consensus to explain these inconsistencies (46–48). Additionally, CS as an environmental factor induces dysbiosis of intestinal microbiota (21, 22). Currently, the effects of smoking on the maternal gut microbiome during pregnancy are not fully characterized, underscoring the need for further research to clarify the role of the gut microbiome during this critical period. Given its modifiability (49), the gut microbiome presents an appealing target for interventions aimed at preventing diseases in pregnant mothers exposed to air pollution, like ETS.

In this study, alpha diversity was not affected by pregnancy in the air-exposed groups, nor by CS exposure in non-pregnant groups. However, CS exposure resulted in a decrease in alpha diversity in pregnant dams, as evidenced by a significant reduction in the number of observed species and the ACE index. This contrasts with a healthy gut microbiome during pregnancy, which is typically characterized by increased alpha diversity (50). Beta diversity was unaffected by pregnancy. CS exposure showed a trend toward affecting beta diversity in non-pregnant mice, aligning with findings from clinical and animal studies where CS had minimal impact (51–53). However, in pregnant mice, CS exposure did significantly decrease beta diversity, as evidenced by reduced Bray-Curtis index values. These changes were confirmed by ANOSIM and MRPP analyses, comparing to both air-exposed pregnant mice and CS-exposed non-pregnant mice. During the fetus development in the third trimester, the total maternal gut microbiota load increased, and the beta diversity among pregnant women increased (48, 54), whereas in our study, CS exposure decreased the beta diversity in pregnant dams. Therefore, we speculate that there are more structural differences in the composition of gut microbiota in the CS-exposed pregnant group.

According to Lefse output, *Cyanobacteria* was one of the distinguishing taxa in pregnant dams exposed to air, but there is insufficient data about its role during pregnancy (55). *Eubacteriaceae* was enriched in non-pregnant CS-exposed mice, similar to previous research of our group (24), which is generally in line with the intestinal microbiome of COPD patients, where the primary risk factors include the inhalation of CS, air pollution or other noxious particles (56). *Turicibacter*, a member of the *Firmicutes* phylum, was the distinguishing taxon in CS-exposed pregnant dams, with its abundance increased in this group compared to CS-exposed non-pregnant mice. During normal pregnancy in both mice and human, as observed in the air-exposed pregnant mice in this study, *Turicibacter* levels typically remain stable or decrease (57, 58). However, in the gut of CS-exposed mice, *Turicibacter* abundance progressively increased, becoming significant after 4 and 5 months of exposure (59). *Turicibacter*, which is enriched in murine colorectal cancer (60), promotes pro-inflammatory effects, while IL-22 treatment, which boosts anti-inflammatory responses, significantly depletes *Turicibacter*, suggesting its involvement in inflammation (61).

One underlying mechanism by which changes in the maternal microbiome influence maternal health and fetal development is the alteration in metabolite production, particularly SCFA produced through microbial fermentation (62). In pregnant air-exposed dams, levels of all cecal SCFA increased compared to non-pregnant air-exposed dams. However, in rat models, only cecal propionate (63), or cecal acetic and propionic acids (64), were increased in the pregnant group compared to the non-pregnant group. The increased SCFA production levels from pregnancy to lactation (as observed in our model) supports the idea of an increase demand for SCFA to support maternal energy requirements, benefiting fetal growth and development (65, 66). CS exposure did not significantly affect cecal SCFA levels in non-pregnant mice, consistent with findings from a previous study conducted by our group on CS exposure in mice (23). However, other clinical and animal evidence indicates that smoking may alter the abundance and function of SCFA-producing bacteria (25). Interestingly, CS exposure did reduce the concentrations of cecal acetate, propionate, valeric, and iso-valeric acid in pregnant dams, similar to the reduction observed in cecal SCFA in pregnant rats exposed to nicotine (63, 64). The impact on the ability of the gut microbiota to metabolize SCFA may vary depending on the specific microbial strains present (67), as well as the smoking dose and duration of exposure (23, 25). These results demonstrate that the combination of pregnancy and CS exposure impacts not only the composition of the microbiota but also its functional capacity, a change that cannot be fully attributed to either CS exposure or pregnancy alone.

SCFA are important immunomodulators in the host (67, 68) with well-documented anti-inflammatory properties at physiological levels. An *in-vitro* model using neutrophils isolated from human blood demonstrated that high concentrations of acetate and butyrate significantly inhibited neutrophil migration compared to control conditions (69). Therefore, a possible explanation for the higher levels of neutrophils in the BALF of pregnant dams exposed to CS could be a reduction in cecal SCFA. Acetate treatment protected against respiratory syncytial virus as decreased the number of macrophages and neutrophils through a G-protein coupled receptor 43 (GPR43)-type 1 interferon response in the BALF of mice (68). Additionally, SCFAs may not directly impact neutrophil migration but could instead influence neutrophil function, such as impairing phagocytic capacity and the production of inflammatory molecules (29, 68). However, a key limitation of our study is the absence of a mechanistic intervention to confirm the role of SCFA in modulating pulmonary immune responses in CS-exposed pregnant dams. Future studies should investigate whether SCFA supplementation can alter lung transcriptomic profiles and neutrophil infiltration.

In addition to SCFA-mediated mechanisms, the pulmonary microbiota may also contribute to the exacerbated neutrophil responses in murine lungs during pregnancy, as both pregnancy (70) and CS exposure (53) have been independently shown to alter the composition and diversity of the lung microbiota in mice. These changes may influence immune responses, including neutrophil activity and cytokine signaling. Further research is needed to elucidate how CS exposure during pregnancy influence the pulmonary microbiome and whether these microbiota-driven effects impact inflammatory processes.

## 5 Conclusion

In conclusion, this study underscores that pregnancy exacerbates neutrophil-driven inflammatory responses in the lungs following CS exposure. Both pregnancy and CS exposure induced distinct transcriptomic changes in the lungs. While pregnancy in air-exposed mice primarily affected genes related to cell division and mitosis, CS exposure predominantly influenced immune response pathways, with neutrophil chemotaxis emerging as the dominant pathway in CS-exposed pregnant dams. This was accompanied by an increased neutrophil count and upregulation of neutrophil chemotaxis-associated genes such as CXCL1, S100A8, and S100A9, supported by elevated protein levels in the BALF. Furthermore, pregnancy was associated with higher SCFA levels in air-exposed dams, whereas CS exposure altered the gut microbiota composition, evidenced by reduced alpha and beta diversity, decreased SCFA levels in pregnant dams. These findings highlight the heightened vulnerability of pregnant dams to CS-induced pulmonary inflammation and gut microbiota dysbiosis, pointing to a potential gut-lung axis interaction that warrants further investigation for its implications on maternal and fetal health.

## Data Availability

The original contributions presented in the study are included in the article/Supplementary Material. The datasets presented in this study can be found in the following online repositories: GEO (accession number GSE302905) and SRA (accession number PRJNA1285318). Further inquiries can be directed to the corresponding author.

## Supplementary material

Supplementary Table 1 Effects of CS exposure on spleen-to-body weight ratio. Values represent the mean (CS-exposed group n = 36, air-exposed group n = 24 mice/group). Data are presented as mean ± SEM; statistical significance was determined using Student t-test (*p < 0.05, ****p < 0.0001 compared to air control).

Supplementary Table 2 Differentially expressed genes in lung tissues of pregnant and non-pregnant mice exposed to CS and air.

Supplementary Figure 1
**(A)** Hierarchical clustering heatmap showing z-scores for the 20 upregulated and 22 downregulated DEGs (padj < 0.05, |log2(fold-change)| > 1) comparing CS-exposed pregnant mice to CS-exposed non-pregnant mice. Red indicates up-regulation, and blue indicates down-regulation. **(B)** The top 20 molecular function pathways from the GO enrichment analysis of DEGs in lung tissue of CS-exposed pregnant and non-pregnant mice are depicted in dot plots. n = 3–4 mice per group (BALF subset).

Supplementary Figure 2 Representative images of BALF cells from four experimental groups, prepared by cytospin and stained with Diff-Quik™. Differential cell counts were performed to identify total cells, macrophages, neutrophils, and lymphocytes based on morphological criteria. Red arrows indicate differences in neutrophil abundance between CS non-pregnant and CS pregnant groups. Note that the cytospin images show cell type distribution but do not reflect changes in total BALF cell numbers. n = 6–10 mice/group (BALF subset).

Supplementary Figure 3 T cell population in the lungs and CXCL2 in the BALF of pregnant and non-pregnant mice exposed to air or CS. Lung cell suspensions were analyzed for the percentage of **(A)** Treg (CD25+, Foxp3+ CD4+), **(B)** Th1 (Tbet+, CXCR3+ CD4+ cells), **(C)** Th2 (T1ST2+, GATA3+ CD4+) by flow cytometry, **(D)** CXCL2, and **(E)** IL-17A levels in the BALF were measured by ELISA. Data are presented as mean ± SEM; n = 11–19 mice/group from both subsets. Statistical significance of differences was evaluated using a two-way ANOVA and *post hoc* Bonferroni’s multiple comparisons test.

Supplementary Figure 4 Impact of CS exposure on microbiota diversity in the feces of pregnant and non-pregnant mice. Alpha diversity is depicted as **(A)** Shannon index and **(B)** Simpson index. Beta diversity is depicted as **(C)** ANOSIM which is a distance-based method and is calculated based on Bray-Curtis dissimilarity. R value > 0 indicates that the difference between groups is greater than the difference within the group. Data are presented as mean ± SEM; n = 10–12 mice/group from both subsets. *p < 0.05, **p < 0.01, and ****p < 0.0001, as determined by two-way ANOVA followed by Bonferroni’s multiple comparisons test. ANP: air non-pregnant, AP: air pregnant, SNP: smoke non-pregnant, SP: smoke pregnant.

Supplementary Figure 5 Impact of CS exposure on microbiota composition in the feces of pregnant and non-pregnant mice. **(A)** The relative abundance of the top 10 most prevalent microbiota at the genus level is displayed. The top 10 predominant genera observed across the experimental groups, in descending order, included Alistipes, Barnesiella, Turicibacter, Romboutsia, Clostridium XIVa, Odoribacter, Oscillibacter, Bacteroides, Clostridium-sensu-stricto, and Acetanifactor. Cladogram of linear discriminant analysis (LDA) effect size (LEfSe) analysis of microbial abundance from phylum to genus level **(B)**; LDA scores of the degree of differentiation between air- and cigarette smoke-exposed mice **(C)**. Data are presented as mean ± SEM; n = 10–12 mice/group from both subsets. The statistical significance of differences was evaluated using a two-way ANOVA and *post hoc* Bonferroni’s multiple comparisons test. ANP: air non-pregnant, AP: air pregnant, SNP: smoke non-pregnant, SP: smoke pregnant.

Supplementary Figure 6 CS exposure did not lead to changes in intestinal permeability and did not alter fecal IgA, fecal and ileal S100A8/A9 levels in pregnant and non-pregnant mice. **(A)** The serum levels of FITC-dextran, a measure of gut permeability (n = 5-9), **(B)** fecal IgA (n = 8-10), **(C)** fecal S100A8/A9 (n = 8-10), and **(D)** ileal S100A8/A9 (n = 11-19) were measured. Data are presented as mean ± SEM; *p < 0.05, as determined by two-way ANOVA followed by Bonferroni’s multiple comparisons test.

Supplementary Figure 7 CS exposure did not significantly impact the concentrations of **(A)** total SCFAs, including **(B)** acetic acid, **(C)** propionic acid, **(D)** butyric acid, and **(E)** iso-butyric acid measured in serum of pregnant and non-pregnant dams. Data are presented as mean ± SEM; n = 11–19 mice/group from both subsets. The statistical significance of differences was evaluated using a two-way ANOVA and *post hoc* Bonferroni’s multiple comparisons test.

Supplementary Figure 8 Gating strategy for FACS analysis of lung cells from both pregnant and non-pregnant dams exposed to CS or air, performed using FlowLogic software.
