# Supplementary material for: Pregnancy exacerbates neutrophil responses in murine lungs and alters gut microbiota composition after cigarette smoke exposure
Source: Front Immunol. 2025 Aug 11;16:1590290. doi: 10.3389/fimmu.2025.1590290 (PMC12375470; doi:10.3389/fimmu.2025.1590290)
Supplement: Supplementary Table 1 — Effects of CS exposure on spleen-to-body weight ratio. Values represent the mean (CS-exposed group n = 36, air-exposed group n = 24 mice/group). Data are presented as mean ± SEM; statistical significance was determined using Student t-test (*p < 0.05, ****p < 0.0001 compared to air control). [file Supplementaryfile1.pdf]

Supplementary data

Supplementary Table 1. Effects of CS exposure on spleen-to-body weight ratio.

	**Experimental groups**		**p value**
	Air	CS	
**Spleen weight/body weight ratio**	0.0041 ± 0.0004	0.0039 ± 0.0002*	0.01
**Body weight (gr)**	24.97 ± 3.21	21.53 ± 2.49****	0.0001
**Spleen weight (gr)**	0.1 ± 0.016	0.08 ± 0.011****	0.0001

Values represent the mean (CS-exposed group n = 36, air-exposed group n = 24 mice/group).
Data are presented as mean ± SEM; statistical significance was determined using Student t-test (*p < 0.05, ****p < 0.0001 compared to air control).

Supplementary Table 2. Differentially expressed genes in lung tissues of pregnant and non-pregnant mice exposed to CS and air

Regulation	Gene ID	Gene symbol	Type	AP vs ANP FC	Qv
Up	1.01E+08	'Kifc1'	mRNA	1.55	0.015195
Up	104732	'Tedc1'	mRNA	1.06	0.005455
Up	107995	'Cdc20'	mRNA	1.28	0.000001
Up	108000	'Cenpf'	mRNA	1.65	0.000002
Up	108907	'Nusap1'	mRNA	1.34	0.002940
Up	108912	'Cdca2'	mRNA	1.63	0.003310
Up	109212	'Pimreg'	mRNA	2.17	0.000149
Up	110033	'Kif22'	mRNA	1.55	0.001925
Up	110749	'Chaf1b'	mRNA	1.01	0.003933
Up	11799	'Birc5'	mRNA	1.41	0.000182
Up	1.19E+08	'LOC118567439'	mRNA	1.41	0.032744
Up	12235	'Bub1'	mRNA	1.53	0.007667
Up	12236	'Bub1b'	mRNA	1.02	0.019265
Up	12316	'Aspm'	mRNA	1.66	0.001046
Up	12428	'Ccna2'	mRNA	1.22	0.003346
Up	12449	'Ccnf'	mRNA	1.23	0.012314
Up	12534	'Cdk1'	mRNA	1.66	0.000001
Up	13605	'Ect2'	mRNA	1.17	0.002765
Up	14793	'Cdca3'	mRNA	1.29	0.011222
Up	15366	'Hmmr'	mRNA	1.70	0.024429
Up	16551	'Kif11'	mRNA	1.20	0.015588
Up	17215	'Mcm3'	mRNA	1.06	0.000520
Up	17218	'Mcm5'	mRNA	1.45	0.000520
Up	17219	'Mcm6'	mRNA	1.27	0.000004
Up	17279	'Melk'	mRNA	2.26	0.002406
Up	17345	'Mki67'	mRNA	1.50	0.000001
Up	18140	'Uhrf1'	mRNA	1.72	0.000000
Up	18817	'Plk1'	mRNA	1.13	0.004256

Regulation	Gene ID	Gene symbol	Type	AP vs ANP FC	Qv
Up	19348	'Kif20a'	mRNA	1.77	0.000968
Up	20135	'Rrm2'	mRNA	1.28	0.000149
Up	20558	'Slfn4'	mRNA	1.05	0.001826
Up	208084	'Pif1'	mRNA	2.55	0.033038
Up	208628	'Kntc1'	mRNA	1.71	0.003473
Up	209003	'Rbmx2'	mRNA	1.11	0.046955
Up	209737	'Kif15'	mRNA	1.46	0.009666
Up	21877	'Tk1'	mRNA	1.13	0.000608
Up	21973	'Top2a'	mRNA	1.28	0.000002
Up	228482	'Arhgap11a'	mRNA	1.15	0.002940
Up	233406	'Prc1'	mRNA	1.43	0.000002
Up	234258	'Neil3'	mRNA	1.74	0.021264
Up	234396	'Ankle1'	mRNA	2.13	0.004034
Up	237877	'Atad5'	mRNA	1.29	0.004943
Up	237886	'Slfn9'	mRNA	1.41	0.000828
Up	241275	'Noxa1'	mRNA	1.05	0.004229
Up	26934	'Racgap1'	mRNA	1.20	0.000008
Up	269582	'Clspn'	mRNA	1.86	0.004274
Up	27221	'Chaf1a'	mRNA	1.19	0.001445
Up	30939	'Pttg1'	mRNA	1.20	0.001826
Up	404710	'Iqgap3'	mRNA	1.82	0.000001
Up	51944	'Knstrn'	mRNA	1.51	0.000069
Up	52033	'Pbk'	mRNA	2.30	0.000004
Up	52276	'Cdca8'	mRNA	1.53	0.015341
Up	54141	'Spag5'	mRNA	1.37	0.020029
Up	60530	'Fignl1'	mRNA	1.66	0.006570
Up	66442	'Spc25'	mRNA	1.55	0.011511
Up	66977	'Nuf2'	mRNA	1.40	0.017274
Up	67052	'Ndc80'	mRNA	2.12	0.012863
Up	67629	'Spc24'	mRNA	1.37	0.017274
Up	68026	'Pclaf'	mRNA	1.20	0.011311
Up	68612	'Ube2c'	mRNA	1.48	0.000161
Up	69716	'Trip13'	mRNA	1.69	0.045126
Up	70024	'Mcm10'	mRNA	1.72	0.004943
Up	70218	'Kif18b'	mRNA	1.94	0.002909
Up	70466	'Ckap2l'	mRNA	1.53	0.000029
Up	72119	'Tpx2'	mRNA	1.37	0.000047
Up	74107	'Cep55'	mRNA	1.44	0.032896
Up	76131	'Depdc1a'	mRNA	2.28	0.039936
Up	76464	'Knl1'	mRNA	1.74	0.011113
Up	76843	'Dtl'	mRNA	1.70	0.003504
Up	78733	'Troap'	mRNA	2.04	0.011557
Up	80986	'Ckap2'	mRNA	1.65	0.004288
Up	99899	'Ifi44'	mRNA	1.10	0.000938

Regulation	Gene ID	Gene symbol	Type	AP vs ANP FC	Qv
Down	11567	'Avil'	mRNA	-1.44	0.008127
Down	118568634	'LOC118568634'	mRNA	-1.33	0.006763
Down	12962	'Crybb3'	mRNA	-1.33	0.000460
Down	15458	'Hpx'	mRNA	-1.17	0.008127
Down	15505	'Hsph1'	mRNA	-1.26	0.026050
Down	15511	'Hspa1b'	mRNA	-2.48	0.002805
Down	16874	'Lhx6'	mRNA	-1.30	0.015529
Down	193740	'Hspa1a'	mRNA	-2.12	0.032744
Down	215493	'A3galt2'	mRNA	-1.85	0.010021
Down	225642	'Grp'	mRNA	-1.14	0.003462
Down	244723	'Olfm2'	mRNA	-1.16	0.010021

Supplementary Table 2. Continue

Regulation	Gene ID	Gene symbol	Type	SNP vs ANP FC	Qv
Up	100034251	'Wfdc17'	mRNA	3.08	4.47E-24
Up	100034748	'A930017K11Rik'	mRNA	3.32	0.007042
Up	100038909	'Gm14548'	mRNA	1.76	9.29E-08
Up	100038947	'Sirpb1c'	mRNA	2.04	3.80E-07
Up	100041146	'Gm15448'	mRNA	5.18	0.002032
Up	100502766	'Kifc1'	mRNA	1.32	0.02188
Up	102680	'Slc6a20a'	mRNA	1.39	3.92E-04
Up	102920	'Cenpi'	mRNA	2.09	9.05E-04
Up	104183	'Chil4'	mRNA	3.80	0.015576
Up	105244829	'Gm40365'	mRNA	5.31	0.017986
Up	108000	'Cenpf'	mRNA	1.12	0.002169
Up	108078	'Olr1'	mRNA	2.02	3.10E-10
Up	108723	'Card11'	mRNA	1.44	2.38E-09
Up	108907	'Nusap1'	mRNA	1.18	0.002399
Up	108912	'Cdca2'	mRNA	1.47	0.03183
Up	109222	'Rarres1'	mRNA	2.40	1.80E-07
Up	109225	'Ms4a7'	mRNA	1.24	0.00717
Up	109245	'Lrrc39'	mRNA	2.69	0.023006
Up	110033	'Kif22'	mRNA	1.28	0.009337
Up	110895	'Slc9a4'	mRNA	1.42	5.73E-08
Up	110956	'D17H6S56E-5'	mRNA	1.03	5.62E-10
Up	11307	'Abcg1'	mRNA	1.24	1.72E-07
Up	11433	'Acp5'	mRNA	1.87	3.46E-23
Up	114644	'Slc13a3'	mRNA	1.85	0.005955
Up	114873	'Dscaml1'	mRNA	2.13	0.02379
Up	11501	'Adam8'	mRNA	1.80	0.002898
Up	11624	'Ahrr'	mRNA	1.43	0.045626
Up	11676	'Aldoc'	mRNA	1.01	1.72E-04
Up	11689	'Alox5'	mRNA	1.65	2.48E-12

Regulation	Gene ID	Gene symbol	Type	SNP vs ANP FC	Qv
Up	11799	'Birc5'	mRNA	1.23	0.002757
Up	11828	'Aqp3'	mRNA	2.66	3.19E-04
Up	118567439	'LOC118567439'	mRNA	1.27	0.030566
Up	118568653	'LOC118568653'	mRNA	5.88	0.004241
Up	11864	'Arnt2'	mRNA	1.85	7.91E-04
Up	11890	'Asgr2'	mRNA	1.29	0.047006
Up	11988	'Slc7a2'	mRNA	1.08	2.06E-08
Up	12044	'Bcl2a1a'	mRNA	1.20	3.57E-06
Up	12045	'Bcl2a1b'	mRNA	1.27	2.46E-06
Up	12046	'Bcl2a1c'	mRNA	2.61	0.008592
Up	12047	'Bcl2a1d'	mRNA	1.19	2.46E-06
Up	12229	'Btk'	mRNA	1.05	2.71E-05
Up	12236	'Bub1b'	mRNA	1.31	3.32E-04
Up	12449	'Ccnf'	mRNA	1.33	2.38E-04
Up	12475	'Cd14'	mRNA	1.03	5.06E-06
Up	12481	'Cd2'	mRNA	1.38	7.65E-09
Up	12483	'Cd22'	mRNA	1.80	1.33E-08
Up	12489	'Cd33'	mRNA	1.38	7.76E-06
Up	12514	'Cd68'	mRNA	2.52	6.64E-34
Up	12523	'Cd84'	mRNA	1.11	2.92E-05
Up	12534	'Cdk1'	mRNA	1.37	1.85E-04
Up	12642	'Ch25h'	mRNA	2.77	3.96E-24
Up	12655	'Chil3'	mRNA	3.49	1.02E-10
Up	12683	'Cidea'	mRNA	1.75	0.040153
Up	12945	'Dmbt1'	mRNA	9.47	0.004724
Up	12952	'Cry1'	mRNA	1.08	1.47E-09
Up	12982	'Csf2ra'	mRNA	1.05	1.81E-06
Up	12983	'Csf2rb'	mRNA	1.38	4.40E-10
Up	12984	'Csf2rb2'	mRNA	1.64	1.41E-10
Up	13033	'Ctsd'	mRNA	1.70	5.89E-16
Up	13038	'Ctsk'	mRNA	2.40	1.74E-35
Up	13040	'Ctss'	mRNA	1.65	9.66E-13
Up	13058	'Cybb'	mRNA	2.02	8.06E-16
Up	13587	'Ear2'	mRNA	1.06	6.34E-05
Up	13733	'Adgre1'	mRNA	1.20	5.04E-06
Up	140493	'Kcnn3'	mRNA	1.66	4.87E-06
Up	140497	'Cd300c2'	mRNA	1.03	4.52E-06
Up	14058	'F10'	mRNA	1.56	1.24E-08
Up	14068	'F7'	mRNA	1.72	5.68E-10
Up	14127	'Fcer1g'	mRNA	1.33	1.68E-10
Up	14131	'Fcgr3'	mRNA	1.47	2.13E-09
Up	14133	'Fcna'	mRNA	1.04	0.048108
Up	14167	'Fgf12'	mRNA	1.92	0.029484
Up	14204	'Il4i1'	mRNA	1.21	6.54E-05

Regulation	Gene ID	Gene symbol	Type	SNP vs ANP FC	Qv
Up	14293	'Fpr1'	mRNA	1.03	0.013265
Up	14311	'Cidec'	mRNA	1.04	0.046278
Up	14347	'Fut7'	mRNA	1.18	0.03397
Up	14727	'Lilr4b'	mRNA	1.60	1.69E-07
Up	14728	'Lilrb4a'	mRNA	1.67	5.64E-09
Up	14793	'Cdca3'	mRNA	1.27	0.002328
Up	14825	'Cxcl1'	mRNA	2.02	4.54E-05
Up	15109	'Hal'	mRNA	1.93	8.57E-09
Up	15118	'Has3'	mRNA	1.13	0.004808
Up	15366	'Hmmr'	mRNA	1.54	0.017255
Up	16173	'Il18'	mRNA	1.09	8.83E-05
Up	16181	'Il1rn'	mRNA	1.52	1.33E-06
Up	16323	'Inhba'	mRNA	2.25	1.01E-07
Up	16411	'Itgax'	mRNA	2.45	7.82E-27
Up	16414	'Itgb2'	mRNA	1.97	8.22E-19
Up	16513	'Kcnj10'	mRNA	2.01	1.14E-05
Up	16514	'Kcnj11'	mRNA	2.03	0.020242
Up	16634	'Klra3'	mRNA	1.65	5.43E-06
Up	16665	'Krt15'	mRNA	3.35	0.019316
Up	16792	'Laptm5'	mRNA	1.22	2.87E-09
Up	16819	'Lcn2'	mRNA	2.10	1.44E-54
Up	16854	'Lgals3'	mRNA	1.16	1.00E-06
Up	17002	'Ltf'	mRNA	5.21	1.56E-04
Up	170638	'Hpcal4'	mRNA	1.18	2.76E-05
Up	170743	'Tlr7'	mRNA	1.17	7.79E-05
Up	17076	'Ly75'	mRNA	1.47	8.35E-10
Up	170779	'Cd209d'	mRNA	1.89	0.012641
Up	170812	'Ahsp'	mRNA	1.79	0.014751
Up	17085	'Ly9'	mRNA	1.32	1.92E-04
Up	171166	'Mcoln3'	mRNA	1.79	4.69E-06
Up	171285	'Havcr2'	mRNA	1.00	0.001546
Up	17136	'Mag'	mRNA	1.06	0.001969
Up	17167	'Marco'	mRNA	2.80	1.06E-04
Up	17218	'Mcm5'	mRNA	1.15	4.17E-04
Up	17219	'Mcm6'	mRNA	1.10	1.87E-06
Up	17279	'Melk'	mRNA	2.12	4.25E-04
Up	17345	'Mki67'	mRNA	1.19	5.67E-05
Up	17381	'Mmp12'	mRNA	4.09	9.94E-91
Up	17474	'Clec4d'	mRNA	1.00	0.004413
Up	17476	'Mpeg1'	mRNA	1.26	2.02E-08
Up	17533	'Mrc1'	mRNA	1.26	5.98E-07
Up	17885	'Myh8'	mRNA	21.21	6.40E-08
Up	17948	'Naip2'	mRNA	1.33	1.17E-05
Up	17969	'Ncf1'	mRNA	1.14	2.50E-07

Regulation	Gene ID	Gene symbol	Type	SNP vs ANP FC	Qv
Up	18140	'Uhrf1'	mRNA	1.54	2.48E-09
Up	18175	'Nrap'	mRNA	1.67	0.011475
Up	18212	'Ntrk2'	mRNA	1.26	6.39E-05
Up	18300	'Oit1'	mRNA	3.53	0.048862
Up	18405	'Orm1'	mRNA	2.92	7.74E-04
Up	18599	'Padi1'	mRNA	2.99	6.12E-04
Up	18722	'Pira1'	mRNA	1.61	3.52E-09
Up	18725	'Pira2'	mRNA	1.67	2.70E-13
Up	18726	'Lilra6'	mRNA	1.67	3.65E-06
Up	18733	'Pirb'	mRNA	1.32	6.06E-08
Up	18796	'Plcb2'	mRNA	1.51	9.54E-12
Up	18807	'Pld3'	mRNA	1.37	1.35E-11
Up	18843	'Bpifa1'	mRNA	8.67	6.26E-06
Up	19016	'Pparg'	mRNA	1.06	4.87E-06
Up	19156	'Psap'	mRNA	1.02	7.30E-13
Up	19222	'Ptgir'	mRNA	1.14	1.72E-04
Up	19348	'Kif20a'	mRNA	1.41	0.003499
Up	19734	'Rgs16'	mRNA	1.57	1.95E-04
Up	19885	'Rorc'	mRNA	1.69	1.36E-18
Up	19894	'Rph3a'	mRNA	6.13	4.47E-05
Up	20135	'Rrm2'	mRNA	1.11	1.16E-04
Up	20210	'Saa3'	mRNA	2.11	1.06E-07
Up	20288	'Msr1'	mRNA	1.92	6.94E-14
Up	20295	'Ccl17'	mRNA	1.35	6.86E-05
Up	20299	'Ccl22'	mRNA	1.67	5.53E-13
Up	20302	'Ccl3'	mRNA	2.03	1.16E-04
Up	20305	'Ccl6'	mRNA	2.39	6.72E-26
Up	20308	'Ccl9'	mRNA	2.00	5.18E-14
Up	20310	'Cxcl2'	mRNA	1.48	0.00514
Up	20321	'Frrs1'	mRNA	1.27	7.02E-04
Up	20375	'Spi1'	mRNA	1.27	1.47E-09
Up	20419	'Shcbp1'	mRNA	1.80	0.005707
Up	20491	'Sla'	mRNA	1.11	1.52E-04
Up	20500	'Slc13a2'	mRNA	3.03	2.54E-04
Up	20537	'Slc5a1'	mRNA	1.02	0.008909
Up	20612	'Siglec1'	mRNA	1.04	2.77E-04
Up	20750	'Spp1'	mRNA	1.94	1.98E-16
Up	20877	'Aurkb'	mRNA	1.35	4.11E-04
Up	20927	'Abcc8'	mRNA	2.34	0.014461
Up	211739	'Vstm2a'	mRNA	1.93	1.91E-04
Up	213409	'Lemd1'	mRNA	2.79	0.017265
Up	21391	'Tbxas1'	mRNA	1.29	3.35E-04
Up	21426	'Tfec'	mRNA	1.21	4.41E-04
Up	214922	'Slc39a2'	mRNA	2.21	9.60E-12

Regulation	Gene ID	Gene symbol	Type	SNP vs ANP FC	Qv
Up	215384	'Fcgbp'	mRNA	1.97	0.028618
Up	217154	'Stac2'	mRNA	1.74	7.57E-07
Up	217169	'Tns4'	mRNA	1.39	0.003971
Up	218454	'Lhfpl2'	mRNA	1.60	1.24E-10
Up	219144	'Arl11'	mRNA	1.51	1.98E-08
Up	21942	'Tnfrsf9'	mRNA	2.21	2.75E-07
Up	21946	'Pglyrp1'	mRNA	1.86	0.029887
Up	21953	'Tnni2'	mRNA	1.68	0.01209
Up	21973	'Top2a'	mRNA	1.01	6.59E-05
Up	22177	'Tyrobp'	mRNA	1.20	3.30E-10
Up	22229	'Ucp3'	mRNA	1.30	1.06E-04
Up	22364	'Vpreb3'	mRNA	1.03	9.51E-05
Up	225471	'Ticam2'	mRNA	1.46	2.72E-05
Up	225638	'Alpk2'	mRNA	2.92	0.049378
Up	226409	'Zranb3'	mRNA	2.03	1.49E-16
Up	227288	'Cxcr1'	mRNA	5.18	1.18E-39
Up	227326	'Gpr55'	mRNA	1.83	6.54E-05
Up	227327	'B3gnt7'	mRNA	2.24	4.94E-15
Up	228482	'Arhgap11a'	mRNA	1.17	9.67E-05
Up	228801	'Bpifb1'	mRNA	6.49	0.0016
Up	230612	'Slc5a9'	mRNA	2.30	0.041501
Up	230899	'Nppa'	mRNA	9.30	0.003595
Up	231691	'Sds'	mRNA	3.95	0.03533
Up	232801	'Lilra5'	mRNA	1.84	2.59E-12
Up	232983	'Cxcl17'	mRNA	1.61	0.042643
Up	233186	'Siglecf'	mRNA	1.75	1.72E-08
Up	233424	'Tmc3'	mRNA	1.39	0.00478
Up	234593	'Ndrg4'	mRNA	1.56	0.040379
Up	234671	'Ces2c'	mRNA	3.01	1.55E-06
Up	236539	'Phgdh'	mRNA	1.41	5.74E-08
Up	237436	'Gas2l3'	mRNA	1.20	0.005858
Up	237560	'Lrrc10'	mRNA	2.70	0.037516
Up	237886	'Slfn9'	mRNA	1.17	6.49E-04
Up	23845	'Clec5a'	mRNA	2.23	1.63E-10
Up	23886	'Gdf15'	mRNA	1.10	0.024164
Up	239393	'Lrp12'	mRNA	1.45	6.75E-10
Up	239849	'Cd200r4'	mRNA	1.60	1.69E-10
Up	23985	'Slc26a4'	mRNA	2.04	9.48E-08
Up	240047	'Mmp25'	mRNA	1.02	0.019958
Up	24088	'Tlr2'	mRNA	1.25	1.34E-07
Up	241274	'Pnpla7'	mRNA	1.08	2.58E-08
Up	241275	'Noxa1'	mRNA	1.09	0.002054
Up	241589	'D430041D05Rik'	mRNA	1.02	0.001004
Up	242125	'Mab21l3'	mRNA	2.23	0.039659

Regulation	Gene ID	Gene symbol	Type	SNP vs ANP FC	Qv
Up	242341	'Atp6v0d2'	mRNA	2.40	7.21E-21
Up	244237	'Tnfrsf26'	mRNA	1.16	0.003499
Up	244238	'Mrgpre'	mRNA	1.07	0.002378
Up	245533	'Awat1'	mRNA	5.53	8.58E-04
Up	246256	'Fcgr4'	mRNA	1.18	1.05E-04
Up	246278	'Cd207'	mRNA	1.00	0.001796
Up	246746	'Cd300lf'	mRNA	1.71	2.02E-10
Up	26464	'Vnn3'	mRNA	1.52	2.85E-04
Up	26888	'Clec4a2'	mRNA	1.29	1.99E-07
Up	26904	'Sh2d1b1'	mRNA	1.48	5.04E-06
Up	269582	'Clspn'	mRNA	1.64	0.005648
Up	269610	'Chd5'	mRNA	1.09	1.36E-05
Up	270685	'Mthfd1l'	mRNA	1.25	1.31E-04
Up	270906	'Prr11'	mRNA	1.38	0.00557
Up	271375	'Cd200r2'	mRNA	7.21	3.46E-08
Up	27221	'Chaf1a'	mRNA	1.05	0.003559
Up	279572	'Tlr13'	mRNA	1.08	1.83E-04
Up	320148	'B430306N03Rik'	mRNA	1.60	4.72E-04
Up	320207	'Pik3r5'	mRNA	1.16	2.14E-05
Up	320827	'Cracd'	mRNA	1.26	2.91E-06
Up	320832	'Sirpb1a'	mRNA	1.57	4.25E-04
Up	327957	'Scimp'	mRNA	1.10	0.001852
Up	330483	'Ceacam16'	mRNA	1.99	4.39E-07
Up	353211	'Prune2'	mRNA	1.14	3.11E-04
Up	380728	'Kcnh4'	mRNA	3.70	6.26E-05
Up	380732	'Milr1'	mRNA	1.10	0.01178
Up	381409	'Cdh26'	mRNA	3.74	0.043521
Up	381484	'Gm5150'	mRNA	1.13	0.00449
Up	381605	'Tbc1d2'	mRNA	1.38	1.31E-10
Up	381836	'Sbk2'	mRNA	2.41	7.32E-07
Up	383787	'Ankrd63'	mRNA	2.26	6.09E-06
Up	404710	'Iqgap3'	mRNA	1.37	3.19E-04
Up	414084	'Tnip3'	mRNA	1.30	0.016889
Up	433470	'AA467197'	mRNA	2.77	0.020949
Up	435626	'Rufy4'	mRNA	2.38	7.42E-12
Up	50778	'Rgs1'	mRNA	2.23	3.66E-07
Up	51944	'Knstrn'	mRNA	1.17	4.85E-04
Up	52033	'Pbk'	mRNA	1.35	0.032359
Up	53419	'Corin'	mRNA	2.03	0.017985
Up	54141	'Spag5'	mRNA	1.28	0.021586
Up	54486	'Hpgds'	mRNA	1.00	0.014204
Up	54635	'Pdgfc'	mRNA	1.38	1.79E-05
Up	56620	'Clec4n'	mRNA	2.42	3.29E-26
Up	56644	'Clec7a'	mRNA	1.66	2.37E-08

Regulation	Gene ID	Gene symbol	Type	SNP vs ANP FC	Qv
Up	56838	'Ccl28'	mRNA	1.82	0.008832
Up	56857	'Slc37a2'	mRNA	1.27	9.29E-06
Up	57262	'Retnla'	mRNA	3.05	7.75E-31
Up	57442	'Kcne3'	mRNA	2.18	6.39E-04
Up	57781	'Cd200r1'	mRNA	1.33	2.84E-05
Up	58217	'Trem1'	mRNA	1.44	0.002113
Up	64099	'Parvg'	mRNA	1.02	2.65E-07
Up	64381	'Ms4a8a'	mRNA	1.73	6.24E-13
Up	65221	'Slc15a3'	mRNA	1.25	3.86E-08
Up	66107	'Wfdc21'	mRNA	2.68	5.89E-12
Up	668101	'Sirpb1b'	mRNA	2.09	0.005195
Up	66901	'Proz'	mRNA	1.83	2.20E-09
Up	66977	'Nuf2'	mRNA	1.15	0.028127
Up	67052	'Ndc80'	mRNA	1.62	0.048108
Up	67133	'Gp2'	mRNA	7.87	0.01109
Up	67426	'Coq8a'	mRNA	1.17	2.62E-04
Up	67717	'Lipf'	mRNA	2.61	4.37E-09
Up	68026	'Pclaf'	mRNA	1.30	3.32E-04
Up	68236	'Gtsf1l'	mRNA	4.62	0.046278
Up	68662	'Scgb3a1'	mRNA	6.21	6.29E-04
Up	68774	'Ms4a6d'	mRNA	1.05	5.06E-06
Up	68891	'Cd177'	mRNA	3.26	3.56E-04
Up	69189	'Mcemp1'	mRNA	1.55	1.69E-08
Up	69769	'Tnfaip8l2'	mRNA	1.11	1.01E-04
Up	70024	'Mcm10'	mRNA	1.58	0.002271
Up	70218	'Kif18b'	mRNA	1.64	0.006298
Up	70789	'Kynu'	mRNA	2.78	1.09E-13
Up	71355	'Col24a1'	mRNA	2.01	0.007069
Up	71816	'Rnf180'	mRNA	1.06	0.002479
Up	71869	'Serpinb12'	mRNA	4.87	0.007841
Up	72043	'Sulf2'	mRNA	1.01	6.99E-09
Up	72119	'Tpx2'	mRNA	1.21	2.51E-04
Up	72169	'Trim29'	mRNA	2.40	2.22E-12
Up	72535	'Aldh1b1'	mRNA	1.94	0.001797
Up	72568	'Lin9'	mRNA	1.45	9.87E-04
Up	73804	'Kif2c'	mRNA	1.46	0.023006
Up	74016	'Phf19'	mRNA	1.03	0.004917
Up	74096	'Hvcn1'	mRNA	1.57	4.73E-14
Up	74152	'Stra6l'	mRNA	2.91	6.04E-14
Up	74183	'Perm1'	mRNA	1.58	0.031236
Up	74191	'P2ry13'	mRNA	1.08	0.003595
Up	74424	'Tmc5'	mRNA	2.61	0.014247
Up	75552	'Paqr9'	mRNA	1.55	0.008832
Up	75677	'Cldn22'	mRNA	3.41	0.030765

Regulation	Gene ID	Gene symbol	Type	SNP vs ANP FC	Qv
Up	76464	'Knl1'	mRNA	1.50	0.005089
Up	76509	'Plet1'	mRNA	1.89	4.34E-63
Up	79201	'Tnfrsf23'	mRNA	1.04	0.012307
Up	79362	'Bhlhe41'	mRNA	1.57	4.33E-15
Up	80782	'Klrb1b'	mRNA	1.08	4.24E-05
Up	80885	'Hcar2'	mRNA	1.59	3.45E-09
Up	83433	'Trem2'	mRNA	2.37	1.54E-19
Up	93695	'Gpnmb'	mRNA	3.58	8.23E-41
Up	96875	'Prg4'	mRNA	1.79	0.030493
Down	1E+08	'Adh6b'	mRNA	-1.71	0.044043
Down	100689	'Spon2'	mRNA	-2.72	0.000013
Down	101401	'Adamts9'	mRNA	-1.05	0.000054
Down	109637	'Upk1a'	mRNA	-1.31	0.000001
Down	11865	'Arntl'	mRNA	-1.77	0.000009
Down	12223	'Btc'	mRNA	-1.02	0.005768
Down	12941	'Pcdha5'	mRNA	-3.25	0.013991
Down	13717	'Eln'	mRNA	-1.50	0.000677
Down	14685	'Gnat1'	mRNA	-1.56	0.013043
Down	14729	'Gp5'	mRNA	-1.29	0.001843
Down	15458	'Hpx'	mRNA	-1.16	0.000896
Down	16009	'Igfbp3'	mRNA	-1.09	0.000002
Down	16625	'Serpina3c'	mRNA	-2.03	0.000016
Down	16948	'Lox'	mRNA	-1.08	0.000000
Down	18054	'Ngp'	mRNA	-2.44	0.000381
Down	20201	'S100a8'	mRNA	-1.10	0.037678
Down	20309	'Cxcl15'	mRNA	-1.15	0.000011
Down	20447	'St6galnac3'	mRNA	-1.27	0.000000
Down	20519	'Slc22a3'	mRNA	-1.08	0.000002
Down	20845	'Star'	mRNA	-1.19	0.016199
Down	215493	'A3galt2'	mRNA	-1.31	0.003214
Down	22355	'Vipr2'	mRNA	-1.23	0.000000
Down	22671	'Rnf112'	mRNA	-2.54	0.044969
Down	233020	'Hipk4'	mRNA	-1.05	0.031236
Down	239337	'Adamts12'	mRNA	-1.01	0.004672
Down	239650	'Ccdc184'	mRNA	-1.15	0.000869
Down	244723	'Olfm2'	mRNA	-1.81	0.000000
Down	245884	'Fam71f2'	mRNA	-1.13	0.000246
Down	26434	'Prnd'	mRNA	-1.18	0.010459
Down	269643	'Ppp2r2c'	mRNA	-1.32	0.029965
Down	276920	'Ccdc42'	mRNA	-1.25	0.000645
Down	30785	'Cttnbp2'	mRNA	-1.05	0.000000
Down	338417	'Scgb1c1'	mRNA	-1.18	0.000081
Down	380694	'Ccnjl'	mRNA	-1.77	0.000639
Down	380702	'Shisa6'	mRNA	-1.60	0.016264

Regulation	Gene ID	Gene symbol	Type	SNP vs ANP FC	Qv
Down	382864	'Colq'	mRNA	-1.71	0.000000
Down	53867	'Col5a3'	mRNA	-1.49	0.000001
Down	54524	'Syt6'	mRNA	-1.04	0.013144
Down	57349	'Ppbp'	mRNA	-1.16	0.000957
Down	574437	'Xlr3b'	mRNA	-2.53	0.010006
Down	60363	'Cldn15'	mRNA	-1.09	0.008832
Down	671232	'Topaz1'	mRNA	-3.16	0.048305
Down	70337	'Iyd'	mRNA	-1.04	0.000149
Down	71566	'Clmp'	mRNA	-1.22	0.000000
Down	73121	'Rflna'	mRNA	-1.19	0.000001
Down	74121	'Acoxl'	mRNA	-1.08	0.003140
Down	93725	'Ear10'	mRNA	-2.55	0.014241

Supplementary Table 2. Continue

Regulation	Gene ID	Gene symbol	Type	SP vs AP FC	Qv
Up	100034251	'Wfdc17'	mRNA	3.93	5.83E-24
Up	100034748	'A930017K11Rik'	mRNA	5.36	0.010108
Up	100038909	'Gm14548'	mRNA	1.42	6.99E-04
Up	100038947	'Sirpb1c'	mRNA	1.25	0.012176
Up	102680	'Slc6a20a'	mRNA	1.45	2.79E-05
Up	108078	'Olr1'	mRNA	2.44	1.21E-37
Up	108723	'Card11'	mRNA	1.37	1.78E-07
Up	109019	'Nabp1'	mRNA	1.21	3.24E-14
Up	109222	'Rarres1'	mRNA	1.82	0.039275
Up	109225	'Ms4a7'	mRNA	1.79	6.91E-06
Up	110895	'Slc9a4'	mRNA	2.03	3.44E-17
Up	11307	'Abcg1'	mRNA	1.21	5.00E-27
Up	11433	'Acp5'	mRNA	1.67	2.52E-14
Up	114644	'Slc13a3'	mRNA	2.12	9.23E-04
Up	11501	'Adam8'	mRNA	1.23	0.002041
Up	11676	'Aldoc'	mRNA	1.15	5.58E-04
Up	11689	'Alox5'	mRNA	1.79	4.07E-08
Up	11690	'Alox5ap'	mRNA	1.04	5.35E-08
Up	117160	'Ttyh2'	mRNA	1.36	2.19E-11
Up	117167	'Steap4'	mRNA	1.29	3.12E-04
Up	11770	'Fabp4'	mRNA	1.18	0.017157
Up	1.19E+08	'LOC118568653'	mRNA	4.50	0.012779
Up	11864	'Arnt2'	mRNA	1.54	0.002701
Up	11890	'Asgr2'	mRNA	1.89	6.64E-05
Up	12044	'Bcl2a1a'	mRNA	1.67	4.50E-14
Up	12045	'Bcl2a1b'	mRNA	1.85	3.85E-22
Up	12047	'Bcl2a1d'	mRNA	2.07	2.60E-29
Up	12260	'C1qb'	mRNA	1.10	7.63E-06
Up	12262	'C1qc'	mRNA	1.07	1.97E-05
Up	12267	'C3ar1'	mRNA	1.20	2.58E-04
Up	12332	'Capg'	mRNA	1.30	1.57E-22
Up	12475	'Cd14'	mRNA	1.70	2.22E-15
Up	12481	'Cd2'	mRNA	1.46	3.54E-13
Up	12483	'Cd22'	mRNA	1.50	1.31E-07
Up	12489	'Cd33'	mRNA	1.11	2.84E-05
Up	12514	'Cd68'	mRNA	2.92	1.30E-78
Up	12519	'Cd80'	mRNA	1.62	4.32E-07
Up	12523	'Cd84'	mRNA	1.08	2.15E-13
Up	12642	'Ch25h'	mRNA	2.88	3.82E-54
Up	12654	'Chil1'	mRNA	1.12	1.97E-10
Up	12655	'Chil3'	mRNA	3.36	7.44E-14
Up	12684	'Cideb'	mRNA	1.29	0.009248

Regulation	Gene ID	Gene symbol	Type	SP vs AP FC	Qv
Up	12768	'Ccr1'	mRNA	1.21	7.46E-06
Up	12865	'Cox7a1'	mRNA	1.32	0.006312
Up	12879	'Cys1'	mRNA	1.34	1.10E-05
Up	12981	'Csf2'	mRNA	1.84	0.002247
Up	12982	'Csf2ra'	mRNA	1.29	2.41E-14
Up	12983	'Csf2rb'	mRNA	1.60	2.21E-19
Up	12984	'Csf2rb2'	mRNA	1.87	7.47E-21
Up	13033	'Ctsd'	mRNA	1.80	1.94E-34
Up	13038	'Ctsk'	mRNA	2.62	6.95E-72
Up	13040	'Ctss'	mRNA	1.50	2.91E-15
Up	13058	'Cybb'	mRNA	1.65	1.36E-17
Up	13587	'Ear2'	mRNA	1.46	2.58E-11
Up	140493	'Kcnn3'	mRNA	1.89	2.78E-06
Up	140497	'Cd300c2'	mRNA	1.29	2.67E-16
Up	14058	'F10'	mRNA	1.83	8.10E-13
Up	14068	'F7'	mRNA	2.05	2.83E-28
Up	140795	'P2ry14'	mRNA	1.14	3.08E-08
Up	14127	'Fcer1g'	mRNA	1.87	3.07E-32
Up	14128	'Fcer2a'	mRNA	1.09	0.012674
Up	14130	'Fcgr2b'	mRNA	1.26	5.13E-09
Up	14131	'Fcgr3'	mRNA	1.77	1.36E-17
Up	14204	'Il4i1'	mRNA	2.05	6.45E-06
Up	14289	'Fpr2'	mRNA	1.39	7.04E-11
Up	14293	'Fpr1'	mRNA	1.12	4.08E-04
Up	14347	'Fut7'	mRNA	1.31	0.017405
Up	14676	'Gna15'	mRNA	1.01	0.003509
Up	14727	'Lilr4b'	mRNA	1.88	5.74E-13
Up	14728	'Lilrb4a'	mRNA	2.02	1.16E-29
Up	14825	'Cxcl1'	mRNA	2.68	0.001978
Up	15109	'Hal'	mRNA	3.14	2.09E-11
Up	16173	'Il18'	mRNA	1.72	5.68E-09
Up	16178	'Il1r2'	mRNA	2.06	0.001506
Up	16181	'Il1rn'	mRNA	2.05	9.20E-11
Up	16323	'Inhba'	mRNA	3.04	4.69E-17
Up	16411	'Itgax'	mRNA	2.51	8.6E-137
Up	16414	'Itgb2'	mRNA	1.80	1.97E-25
Up	16424	'Itih1'	mRNA	1.94	2.21E-05
Up	16427	'Itih4'	mRNA	1.28	1.46E-04
Up	16592	'Fabp5'	mRNA	1.28	7.91E-32
Up	16634	'Klra3'	mRNA	1.61	3.16E-08
Up	16819	'Lcn2'	mRNA	3.09	2.53E-46
Up	16854	'Lgals3'	mRNA	1.70	9.18E-38
Up	17002	'Ltf'	mRNA	3.55	0.036137
Up	170743	'Tlr7'	mRNA	1.05	8.74E-06

Regulation	Gene ID	Gene symbol	Type	SP vs AP FC	Qv
Up	17076	'Ly75'	mRNA	1.18	2.54E-15
Up	170812	'Ahsp'	mRNA	1.70	0.009622
Up	17085	'Ly9'	mRNA	1.26	1.40E-05
Up	171166	'Mcoln3'	mRNA	1.45	7.56E-05
Up	171285	'Havcr2'	mRNA	1.16	5.00E-05
Up	17136	'Mag'	mRNA	1.33	0.002682
Up	17167	'Marco'	mRNA	2.78	8.47E-06
Up	17381	'Mmp12'	mRNA	4.56	1.42E-75
Up	17474	'Clec4d'	mRNA	1.71	2.39E-04
Up	17476	'Mpeg1'	mRNA	1.04	3.57E-09
Up	17533	'Mrc1'	mRNA	1.08	5.25E-19
Up	17916	'Myo1f'	mRNA	1.05	2.45E-11
Up	17948	'Naip2'	mRNA	1.09	0.014309
Up	17969	'Ncf1'	mRNA	1.14	3.20E-10
Up	17970	'Ncf2'	mRNA	1.23	1.62E-09
Up	17972	'Ncf4'	mRNA	1.10	1.03E-07
Up	18037	'Nfkbie'	mRNA	1.03	1.22E-06
Up	18301	'Fxyd5'	mRNA	1.10	3.72E-28
Up	18405	'Orm1'	mRNA	1.99	0.041722
Up	18406	'Orm2'	mRNA	4.73	0.046039
Up	18722	'Pira1'	mRNA	1.37	1.20E-04
Up	18725	'Pira2'	mRNA	1.46	8.14E-09
Up	18726	'Lilra6'	mRNA	1.25	0.001425
Up	18733	'Pirb'	mRNA	1.20	1.63E-09
Up	18768	'Pkib'	mRNA	1.32	1.70E-04
Up	18796	'Plcb2'	mRNA	1.64	2.14E-11
Up	18807	'Pld3'	mRNA	1.55	2.38E-37
Up	18843	'Bpifa1'	mRNA	6.28	0.012484
Up	19222	'Ptgir'	mRNA	1.68	5.19E-09
Up	19734	'Rgs16'	mRNA	1.80	4.12E-06
Up	19885	'Rorc'	mRNA	1.40	3.33E-12
Up	19894	'Rph3a'	mRNA	3.76	0.001151
Up	20210	'Saa3'	mRNA	3.77	1.22E-05
Up	20288	'Msr1'	mRNA	1.86	3.05E-08
Up	20295	'Ccl17'	mRNA	2.24	9.01E-07
Up	20296	'Ccl2'	mRNA	1.86	0.015255
Up	20299	'Ccl22'	mRNA	2.40	4.88E-15
Up	20302	'Ccl3'	mRNA	2.06	4.93E-07
Up	20305	'Ccl6'	mRNA	2.73	1.04E-37
Up	20306	'Ccl7'	mRNA	1.80	0.021288
Up	20308	'Ccl9'	mRNA	2.64	9.03E-38
Up	20310	'Cxcl2'	mRNA	3.09	3.61E-11
Up	20311	'Cxcl5'	mRNA	2.31	1.74E-05
Up	20375	'Spi1'	mRNA	1.47	2.37E-20

Regulation	Gene ID	Gene symbol	Type	SP vs AP FC	Qv
Up	20491	'Sla'	mRNA	1.09	8.48E-05
Up	20568	'Slpi'	mRNA	1.29	7.63E-14
Up	20750	'Spp1'	mRNA	2.47	2.69E-27
Up	207839	'Galnt6'	mRNA	1.07	2.39E-04
Up	211739	'Vstm2a'	mRNA	1.84	0.002493
Up	213409	'Lemd1'	mRNA	3.79	0.001169
Up	21391	'Tbxas1'	mRNA	1.47	5.86E-09
Up	21426	'Tfec'	mRNA	1.47	1.71E-05
Up	214922	'Slc39a2'	mRNA	2.59	1.54E-22
Up	217154	'Stac2'	mRNA	1.48	8.29E-04
Up	217169	'Tns4'	mRNA	1.43	0.008686
Up	217303	'Cd300a'	mRNA	1.17	1.93E-07
Up	217304	'Cd300lb'	mRNA	1.04	0.005571
Up	218454	'Lhfpl2'	mRNA	1.17	3.21E-09
Up	219144	'Arl11'	mRNA	1.80	2.64E-10
Up	21926	'Tnf'	mRNA	1.66	0.005035
Up	21928	'Tnfaip2'	mRNA	1.12	2.85E-07
Up	21942	'Tnfrsf9'	mRNA	1.96	1.34E-04
Up	21946	'Pglyrp1'	mRNA	1.46	0.001863
Up	21953	'Tnni2'	mRNA	1.92	0.002945
Up	22177	'Tyrobp'	mRNA	1.42	3.99E-15
Up	22229	'Ucp3'	mRNA	1.37	5.34E-04
Up	22361	'Vnn1'	mRNA	1.96	2.78E-10
Up	226409	'Zranb3'	mRNA	1.69	8.39E-13
Up	227288	'Cxcr1'	mRNA	4.93	6.53E-35
Up	227326	'Gpr55'	mRNA	1.59	0.002317
Up	227327	'B3gnt7'	mRNA	2.45	3.39E-35
Up	228801	'Bpifb1'	mRNA	4.91	0.025019
Up	230738	'Zc3h12a'	mRNA	1.01	1.85E-05
Up	230899	'Nppa'	mRNA	8.41	0.038766
Up	231805	'Pilra'	mRNA	1.28	2.09E-04
Up	232801	'Lilra5'	mRNA	1.83	7.97E-16
Up	232983	'Cxcl17'	mRNA	1.26	0.001659
Up	233186	'Siglecf'	mRNA	1.85	3.57E-15
Up	233424	'Tmc3'	mRNA	1.62	0.031105
Up	234671	'Ces2c'	mRNA	2.96	7.56E-06
Up	236539	'Phgdh'	mRNA	1.28	4.19E-07
Up	23834	'Cdc6'	mRNA	1.07	0.023001
Up	23845	'Clec5a'	mRNA	1.94	9.97E-08
Up	23886	'Gdf15'	mRNA	1.97	6.26E-08
Up	239393	'Lrp12'	mRNA	1.45	4.65E-15
Up	239849	'Cd200r4'	mRNA	2.01	1.18E-20
Up	23985	'Slc26a4'	mRNA	2.94	1.23E-14
Up	24088	'Tlr2'	mRNA	1.15	1.54E-07

Regulation	Gene ID	Gene symbol	Type	SP vs AP FC	Qv
Up	241274	'Pnpla7'	mRNA	1.05	1.58E-10
Up	242125	'Mab21l3'	mRNA	2.41	6.45E-06
Up	242341	'Atp6v0d2'	mRNA	2.71	5.82E-54
Up	243967	'Ntn5'	mRNA	1.03	0.002196
Up	244237	'Tnfrsf26'	mRNA	1.37	7.96E-05
Up	244238	'Mrgpre'	mRNA	1.07	0.022999
Up	245533	'Awat1'	mRNA	6.84	2.99E-05
Up	246256	'Fcgr4'	mRNA	1.58	2.15E-09
Up	246746	'Cd300lf'	mRNA	1.82	2.05E-17
Up	26464	'Vnn3'	mRNA	1.40	0.008195
Up	26888	'Clec4a2'	mRNA	1.04	0.038337
Up	26904	'Sh2d1b1'	mRNA	1.39	7.61E-07
Up	269610	'Chd5'	mRNA	1.09	0.010676
Up	271375	'Cd200r2'	mRNA	8.08	2.46E-08
Up	277468	'Slc39a12'	mRNA	3.17	0.022778
Up	320148	'B430306N03Rik'	mRNA	1.56	0.001091
Up	320207	'Pik3r5'	mRNA	1.08	1.29E-11
Up	320827	'Cracd'	mRNA	1.27	0.00167
Up	320832	'Sirpb1a'	mRNA	1.28	0.004118
Up	327957	'Scimp'	mRNA	1.29	2.63E-04
Up	330122	'Cxcl3'	mRNA	3.10	4.40E-04
Up	330483	'Ceacam16'	mRNA	2.44	3.16E-08
Up	353211	'Prune2'	mRNA	1.47	3.02E-04
Up	380732	'Milr1'	mRNA	1.27	0.002945
Up	381269	'Mreg'	mRNA	1.14	1.56E-05
Up	381605	'Tbc1d2'	mRNA	1.21	9.41E-05
Up	381836	'Sbk2'	mRNA	2.68	8.24E-05
Up	383787	'Ankrd63'	mRNA	2.74	6.66E-05
Up	414084	'Tnip3'	mRNA	1.43	0.024113
Up	435626	'Rufy4'	mRNA	2.28	2.81E-09
Up	50778	'Rgs1'	mRNA	2.01	1.16E-04
Up	53314	'Batf'	mRNA	1.13	0.003153
Up	54486	'Hpgds'	mRNA	1.13	0.006256
Up	54519	'Apbb1ip'	mRNA	1.12	3.13E-08
Up	56620	'Clec4n'	mRNA	2.59	1.47E-44
Up	56644	'Clec7a'	mRNA	1.93	1.38E-35
Up	57248	'Ly6i'	mRNA	1.02	1.20E-04
Up	57262	'Retnla'	mRNA	3.09	5.97E-24
Up	574404	'Gm14685'	mRNA	3.15	0.034036
Up	57781	'Cd200r1'	mRNA	1.43	1.72E-07
Up	58217	'Trem1'	mRNA	2.03	3.69E-05
Up	613123	'Ugt1a8'	mRNA	21.33	4.76E-06
Up	64099	'Parvg'	mRNA	1.23	1.26E-08
Up	64138	'Ctsz'	mRNA	1.09	1.79E-24

Regulation	Gene ID	Gene symbol	Type	SP vs AP FC	Qv
Up	64381	'Ms4a8a'	mRNA	1.84	6.35E-16
Up	65221	'Slc15a3'	mRNA	1.23	5.85E-06
Up	66107	'Wfdc21'	mRNA	3.24	2.53E-27
Up	66205	'Cd302'	mRNA	1.00	1.22E-05
Up	666907	'Ms4a4a'	mRNA	1.05	0.017796
Up	66901	'Proz'	mRNA	2.23	6.35E-12
Up	67717	'Lipf'	mRNA	3.79	5.14E-12
Up	68396	'Nat8'	mRNA	1.59	0.049693
Up	68662	'Scgb3a1'	mRNA	5.02	0.006255
Up	68774	'Ms4a6d'	mRNA	1.32	1.72E-12
Up	69069	'Tmem273'	mRNA	1.30	2.51E-04
Up	69189	'Mcemp1'	mRNA	1.53	1.88E-17
Up	69769	'Tnfaip8l2'	mRNA	1.19	1.30E-05
Up	70350	'Basp1'	mRNA	1.14	3.13E-04
Up	70789	'Kynu'	mRNA	2.33	1.53E-12
Up	71355	'Col24a1'	mRNA	3.54	8.48E-05
Up	71816	'Rnf180'	mRNA	1.17	0.001834
Up	71893	'Noxo1'	mRNA	1.62	5.34E-10
Up	72042	'Cotl1'	mRNA	1.22	5.27E-07
Up	72169	'Trim29'	mRNA	2.55	1.34E-16
Up	73822	'Mfsd12'	mRNA	1.01	2.51E-13
Up	74096	'Hvcn1'	mRNA	1.75	1.12E-14
Up	74152	'Stra6l'	mRNA	2.83	1.31E-15
Up	74191	'P2ry13'	mRNA	1.36	1.99E-04
Up	75697	'C2cd4b'	mRNA	1.02	5.34E-05
Up	75766	'Dcstamp'	mRNA	3.08	0.013949
Up	76509	'Plet1'	mRNA	1.77	1.68E-34
Up	76905	'Lrg1'	mRNA	1.06	5.03E-05
Up	79201	'Tnfrsf23'	mRNA	1.42	7.78E-04
Up	79362	'Bhlhe41'	mRNA	1.28	2.38E-04
Up	80782	'Klrb1b'	mRNA	1.14	4.70E-04
Up	80885	'Hcar2'	mRNA	2.22	3.71E-32
Up	83433	'Trem2'	mRNA	2.47	2.19E-38
Up	93674	'Nat8f3'	mRNA	1.00	5.45E-04
Up	93695	'Gpnmb'	mRNA	3.61	8.16E-99
Up	98365	'Slamf9'	mRNA	1.10	4.27E-04
Down	100040500	'Gm2808'	mRNA	-1.79875	8.88E-04
Down	100689	'Spon2'	mRNA	-1.57385	1.08E-04
Down	109637	'Upk1a'	mRNA	-1.26	0.048725
Down	11865	'Arntl'	mRNA	-1.44195	0.016208
Down	12069	'Bex2'	mRNA	-1.13423	1.98E-05
Down	12223	'Btc'	mRNA	-1.3767	0.005189
Down	13076	'Cyp1a1'	mRNA	-1.20823	0.002929
Down	13521	'Slc26a2'	mRNA	-1.64253	4.32E-07

Regulation	Gene ID	Gene symbol	Type	SP vs AP FC	Qv
Down	13841	'Epha7'	mRNA	-1.17621	0.005035
Down	14585	'Gfra1'	mRNA	-1.1415	0.017829
Down	14652	'Glp1r'	mRNA	-1.02839	0.045207
Down	16485	'Kcna1'	mRNA	-2.45631	0.015284
Down	16625	'Serpina3c'	mRNA	-1.55218	0.037822
Down	17203	'Mc5r'	mRNA	-2.23477	0.034097
Down	192212	'Prom2'	mRNA	-1.29473	0.028823
Down	20519	'Slc22a3'	mRNA	-1.6006	3.10E-06
Down	21892	'Tll1'	mRNA	-1.2553	0.006225
Down	227394	'Slco4c1'	mRNA	-1.1243	0.025088
Down	230316	'Megf9'	mRNA	-1.20364	2.90E-05
Down	234564	'Ces1f'	mRNA	-1.52589	0.001078
Down	239650	'Ccdc184'	mRNA	-1.08196	0.003257
Down	26875	'Pclo'	mRNA	-1.5321	0.043092
Down	30785	'Cttnbp2'	mRNA	-1.13937	1.08E-04
Down	329482	'Dcdc5'	mRNA	-1.14146	0.013473
Down	380694	'Ccnjl'	mRNA	-1.68076	0.009122
Down	380959	'Alg10b'	mRNA	-1.05276	7.63E-06
Down	382864	'Colq'	mRNA	-1.35899	3.21E-07
Down	442834	'D830031N03Rik'	mRNA	-1.57057	0.016552
Down	53867	'Col5a3'	mRNA	-1.22499	0.003765
Down	56293	'Slc35g3'	mRNA	-1.59693	0.044149
Down	665033	'Col6a5'	mRNA	-1.65909	0.00657
Down	667214	'9930111J21Rik1'	mRNA	-1.08082	0.006803
Down	68312	'Gstm7'	mRNA	-1.1288	0.006034
Down	70337	'Iyd'	mRNA	-1.05587	0.024616
Down	70846	'Ttc6'	mRNA	-1.30161	9.54E-04
Down	73472	'Spata18'	mRNA	-1.92727	1.42E-04
Down	74121	'Acoxl'	mRNA	-1.43565	4.67E-15
Down	94226	'S1pr5'	mRNA	-1.07729	0.030751

Supplementary Table 2. Continue

Regulation	Gene ID	Gene symbol	Type	SP vs SNP FC	Qv
Up	12765	'Cxcr2'	mRNA	1.20	0.029666
Up	12986	'Csf3r'	mRNA	1.26	0.024048
Up	14204	'Il4i1'	mRNA	1.25	0.005966
Up	14825	'Cxcl1'	mRNA	1.33	1.81E-04
Up	16176	'Il1b'	mRNA	1.20	0.006325
Up	18054	'Ngp'	mRNA	1.74	0.015659
Up	20201	'S100a8'	mRNA	1.80	6.38E-05
Up	20202	'S100a9'	mRNA	1.59	0.003821
Up	20210	'Saa3'	mRNA	2.35	8.89E-13
Up	20296	'Ccl2'	mRNA	2.11	0.037215
Up	20310	'Cxcl2'	mRNA	1.30	0.001659
Up	20311	'Cxcl5'	mRNA	1.79	0.014111
Up	20555	'Slfn1'	mRNA	1.10	0.047919
Up	20558	'Slfn4'	mRNA	1.49	2.13E-05
Up	21857	'Timp1'	mRNA	1.17	3.90E-05
Up	21877	'Tk1'	mRNA	1.06	0.035934
Up	242125	'Mab21l3'	mRNA	1.23	0.046469
Up	613123	'Ugt1a8'	mRNA	21.72	7.36E-06
Up	620499	'Gm6158'	mRNA	7.07	4.38E-09
Up	74466	'Mfsd13b'	mRNA	1.24	1.51E-04
Down	100040500	'Gm2808'	mRNA	-1.56	0.035934
Down	118567641	'LOC118567641'	mRNA	-1.15	8.46E-05
Down	118568284	'LOC118568284'	mRNA	-3.25	0.013148
Down	13076	'Cyp1a1'	mRNA	-1.31	0.001659
Down	13521	'Slc26a2'	mRNA	-1.50	0.002117
Down	13714	'Elk4'	mRNA	-1.11	1.38E-04
Down	16656	'Hivep3'	mRNA	-1.11	0.033724
Down	20564	'Slit3'	mRNA	-1.01	6.80E-05
Down	209239	'Gan'	mRNA	-1.41	0.046469
Down	21951	'Tnks'	mRNA	-1.06	8.46E-05
Down	227394	'Slco4c1'	mRNA	-1.36	0.025445
Down	230316	'Megf9'	mRNA	-1.37	0.001142
Down	238803	'Zfp366'	mRNA	-1.79	0.001260
Down	239528	'Ago2'	mRNA	-1.37	7.36E-06
Down	320799	'Zhx3'	mRNA	-1.11	1.28E-06
Down	380959	'Alg10b'	mRNA	-1.03	0.003997

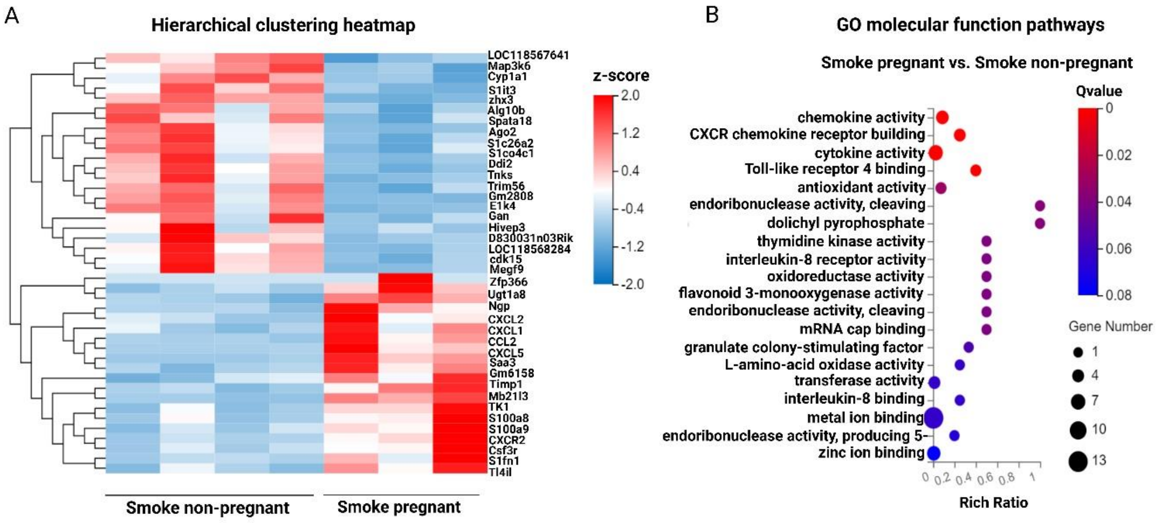

Supplementary Figure 1. (A) Hierarchical clustering heatmap showing z-scores for the 20 upregulated and 22 downregulated DEGs (padj < 0.05, |log2(fold-change)| > 1) comparing CS-exposed pregnant mice to CS-exposed non-pregnant mice. Red indicates up-regulation, and blue indicates down-regulation. (B) The top 20 molecular function pathways from the GO enrichment analysis of DEGs in lung tissue of CS-exposed pregnant and non-pregnant mice are depicted in dot plots. n = 3-4 mice per group (BALF subset).

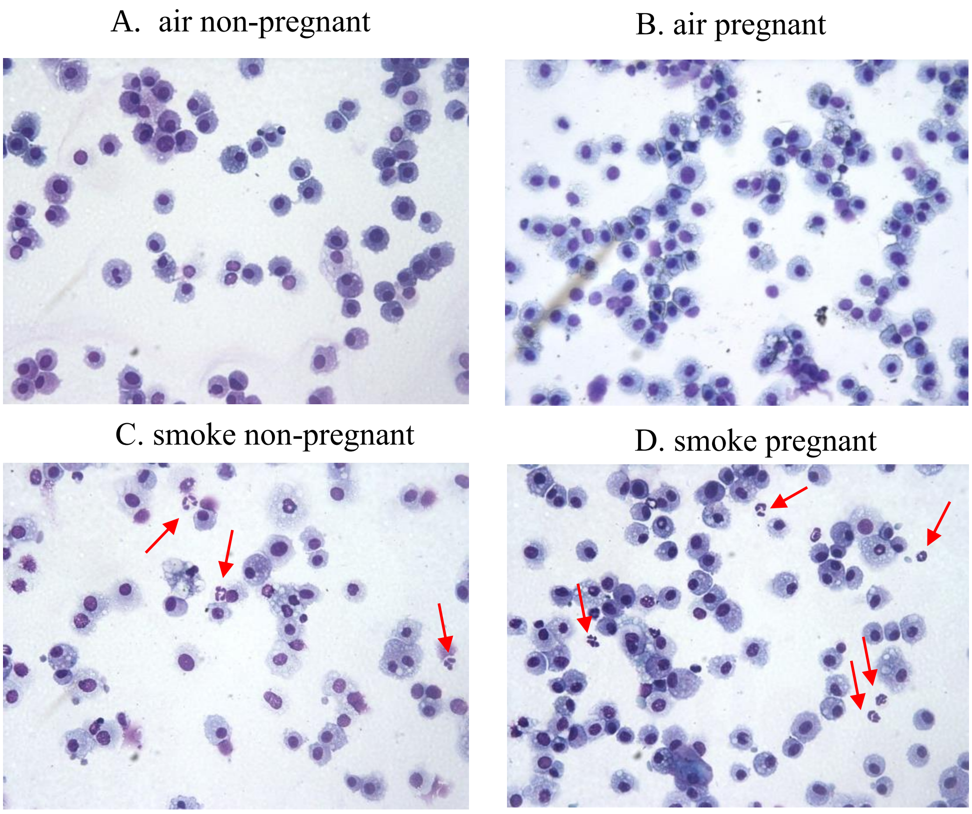

Supplementary Figure 2. Representative images of BALF cells from four experimental groups, prepared by cytospin and stained with Diff-Quik™. Differential cell counts were performed to identify total cells, macrophages, neutrophils, and lymphocytes based on morphological criteria. Red arrows indicate differences in neutrophil abundance between CS non-pregnant and CS pregnant groups. Note that the cytospin images show cell type distribution but do not reflect changes in total BALF cell numbers. n = 6-10 mice/group (BALF subset).

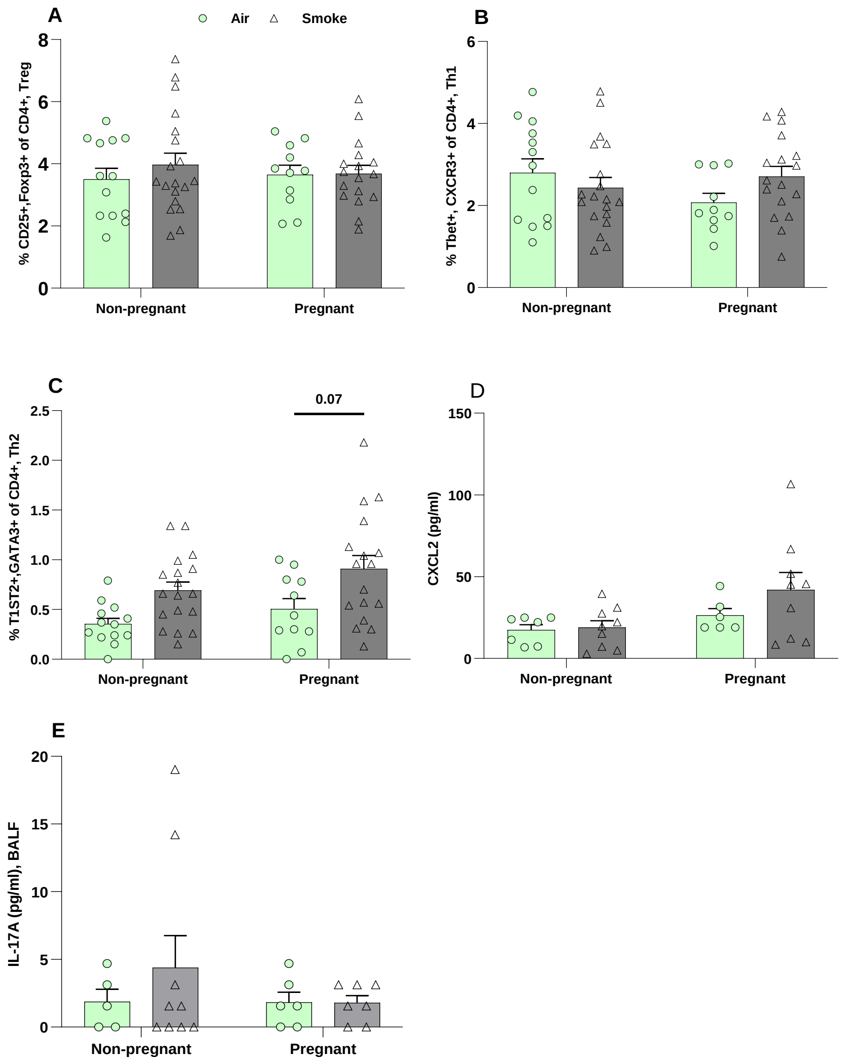

Supplementary Figure 3. T cell population in the lungs and CXCL2 in the BALF of pregnant and non-pregnant mice exposed to air or CS. Lung cell suspensions were analyzed for the percentage of (A) Treg (CD25+, Foxp3+ CD4+), (B) Th1 (Tbet+, CXCR3+ CD4+ cells), (C) Th2 (T1ST2+, GATA3+ CD4+) by flow cytometry, (D) CXCL2, and (E) IL-17A levels in the BALF were measured by ELISA. Data are presented as mean ± SEM; n = 11-19 mice/group from both subsets. Statistical significance of differences was evaluated using a two-way ANOVA and post hoc Bonferroni's multiple comparisons test.

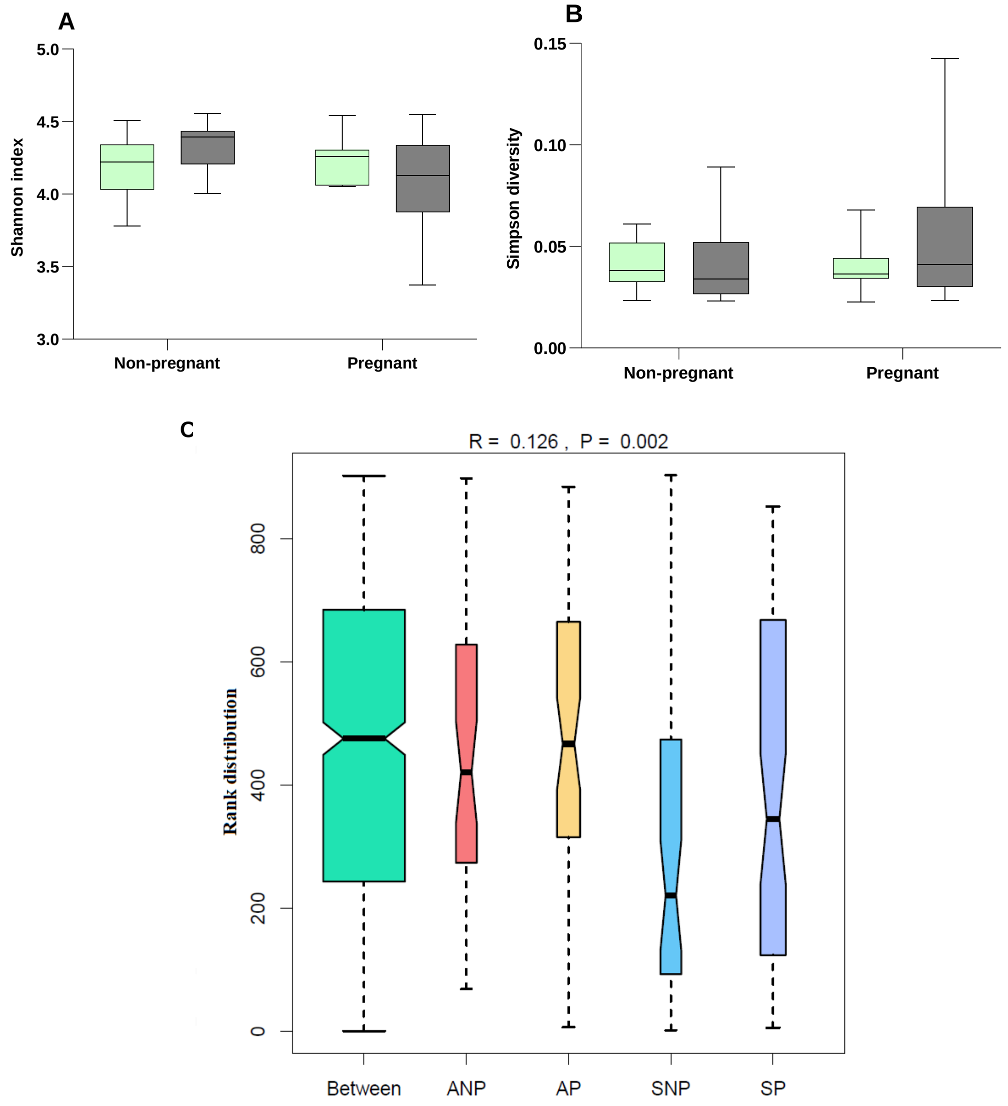

Supplementary Figure 4. Impact of CS exposure on microbiota diversity in the feces of pregnant and non-pregnant mice. Alpha diversity is depicted as (A) Shannon index and (B) Simpson index. Beta diversity is depicted as (C) ANOSIM which is a distance-based method and is calculated based on Bray-Curtis dissimilarity. R value > 0 indicates that the difference between groups is greater than the difference within the group. Data are presented as mean ± SEM; n = 10-12 mice/group from both subsets. *p < 0.05, **p < 0.01, and ****p < 0.0001, as determined by two-way ANOVA followed by Bonferroni's multiple comparisons test. ANP: air non-pregnant, AP: air pregnant, SNP: smoke non-pregnant, SP: smoke pregnant.

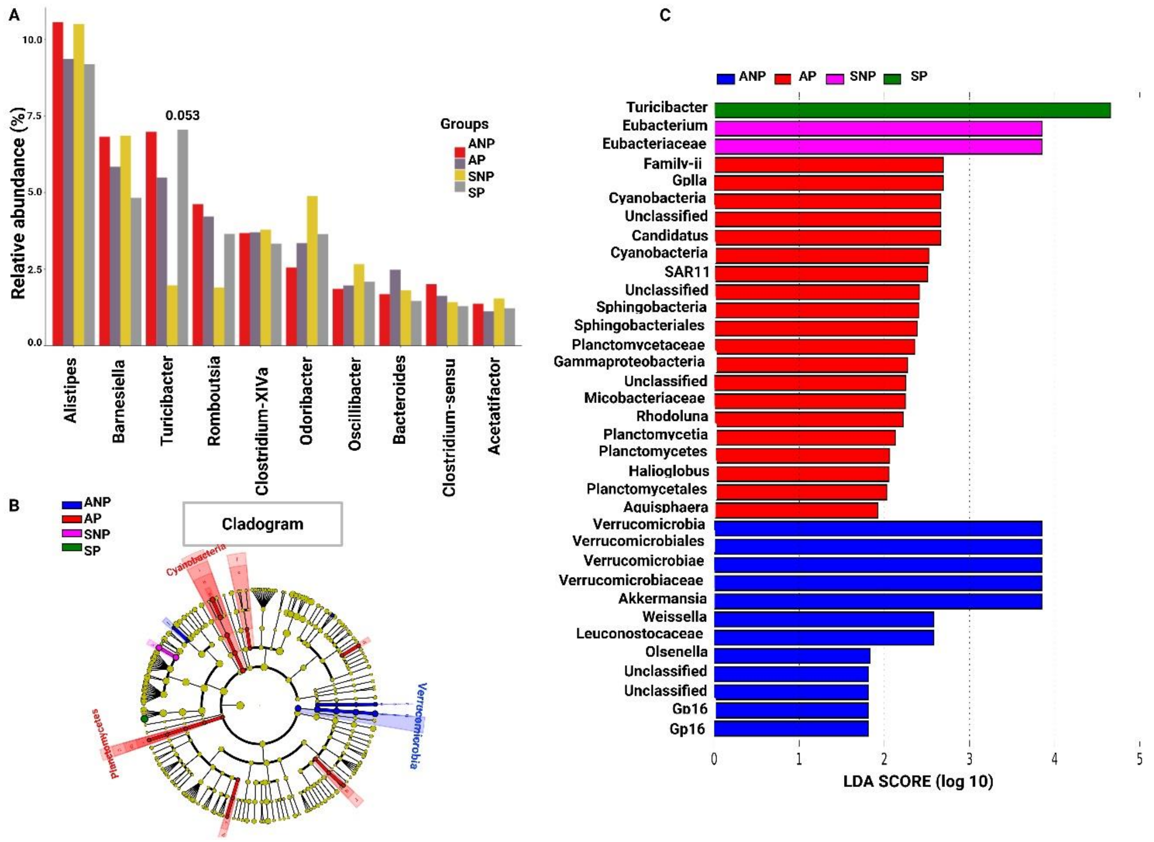

Supplementary Figure 5. Impact of CS exposure on microbiota composition in the feces of pregnant and non-pregnant mice. (A) The relative abundance of the top 10 most prevalent microbiota at the genus level is displayed. The top 10 predominant genera observed across the experimental groups, in descending order, included Alistipes, Barnesiella, Turicibacter, Romboutsia, Clostridium XIVa, Odoribacter, Oscillibacter, Bacteroides, Clostridium-sensu-stricto, and Acetanifactor. Cladogram of linear discriminant analysis (LDA) effect size (LEfSe) analysis of microbial abundance from phylum to genus level (B); LDA scores of the degree of differentiation between air- and cigarette smoke-exposed mice (C). Data are presented as mean ± SEM; n = 10-12 mice/group from both subsets. The statistical significance of differences was evaluated using a two-way ANOVA and post hoc Bonferroni’s multiple comparisons test. ANP: air non-pregnant, AP: air pregnant, SNP: smoke non-pregnant, SP: smoke pregnant.

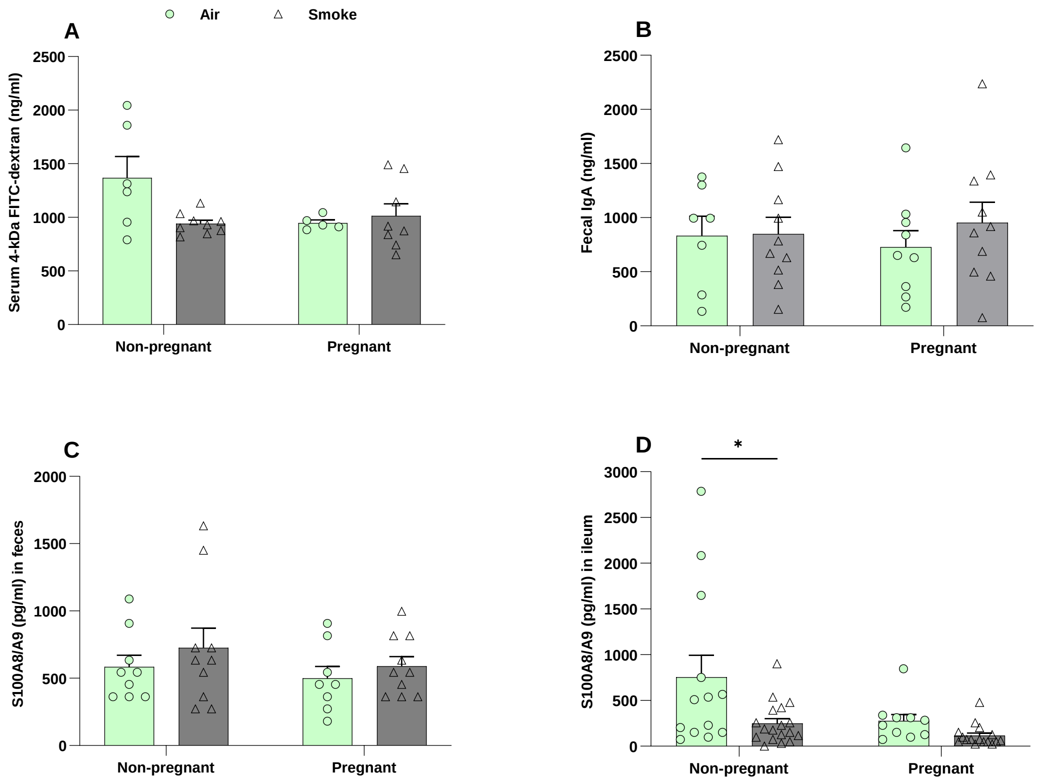

Supplementary Figure 6. CS exposure did not lead to changes in intestinal permeability and did not alter fecal IgA, fecal and ileal S100A8/A9 levels in pregnant and non-pregnant mice. (A) The serum levels of FITC-dextran, a measure of gut permeability (n = 5-9), (B) fecal IgA (n = 8-10), (C) fecal S100A8/A9 (n = 8-10), and (D) ileal S100A8/A9 (n = 11-19) were measured. Data are presented as mean ± SEM; *$p < 0.05$, as determined by two-way ANOVA followed by Bonferroni's multiple comparisons test.

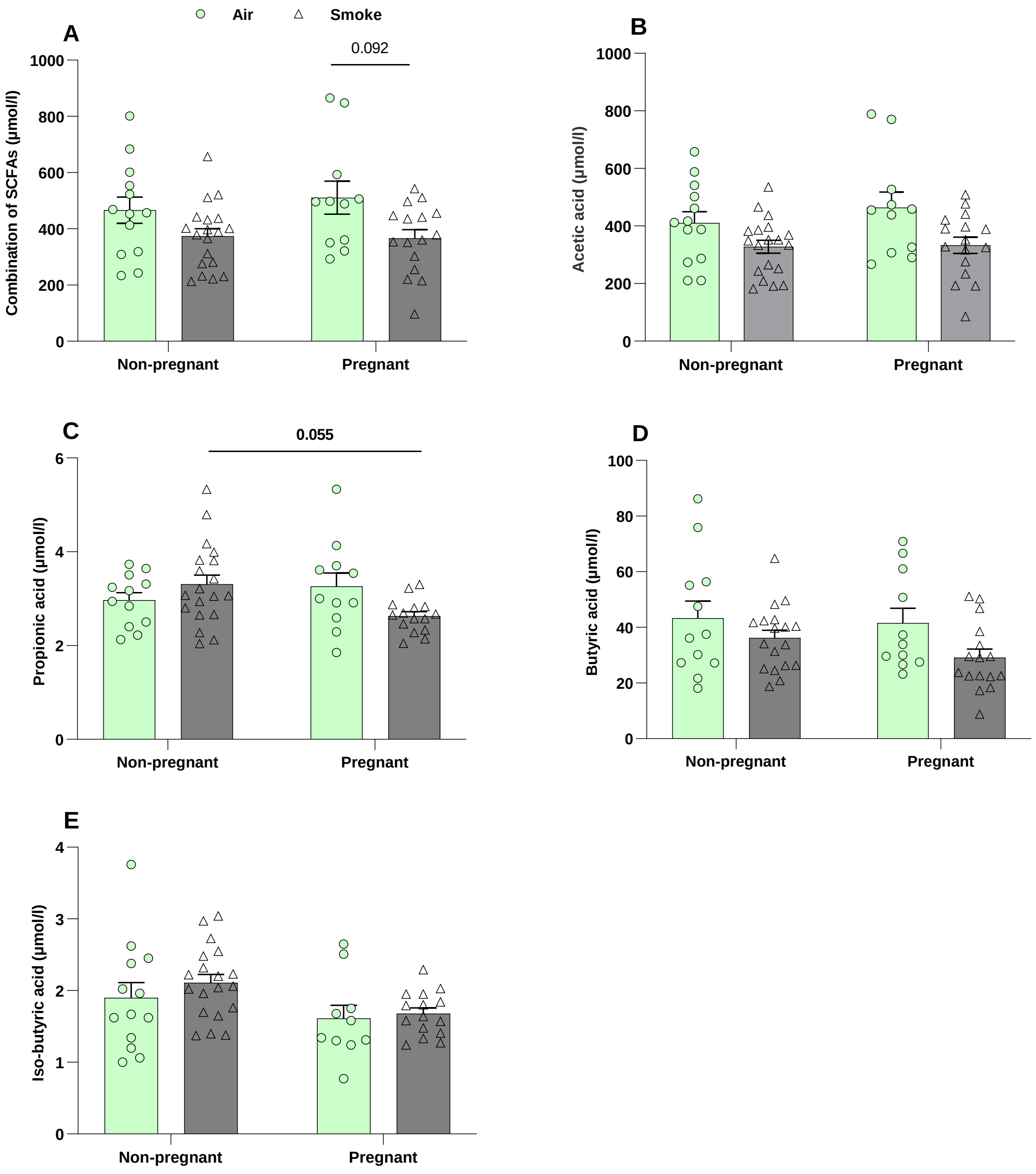

Supplementary Figure 7. CS exposure did not significantly impact the concentrations of (A) total SCFAs, including (B) acetic acid, (C) propionic acid, (D) butyric acid, and (E) iso-butyric acid measured in serum of pregnant and non-pregnant dams. Data are presented as mean ± SEM; n = 11-19 mice/group from both subsets. The statistical significance of differences was evaluated using a two-way ANOVA and post hoc Bonferroni's multiple comparisons test.

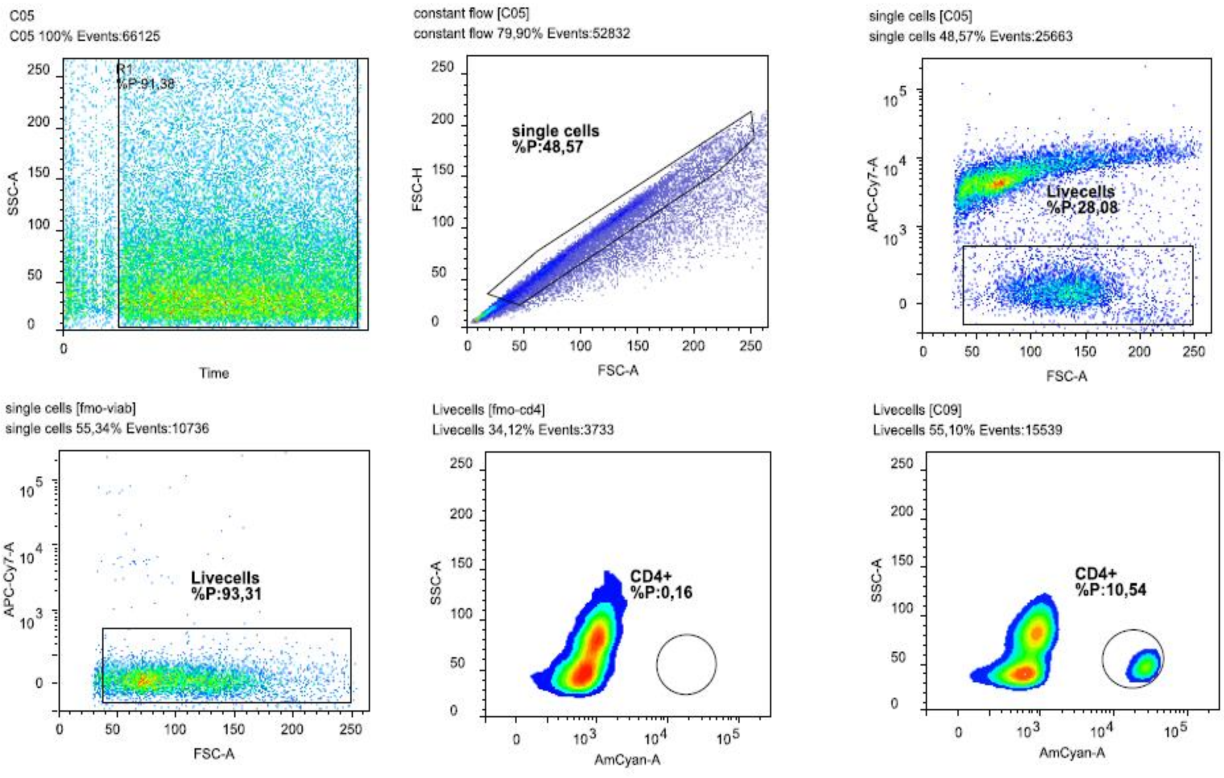

Supplementary Figure 8. Gating strategy for FACS analysis of lung cells from both pregnant and non-pregnant dams exposed to CS or air, performed using FlowLogic software.
